# A Milk Extracellular Vesicle‐Based Nanoplatform Enhances Combination Therapy Against Multidrug‐Resistant Bacterial Infections

**DOI:** 10.1002/advs.202406496

**Published:** 2024-12-25

**Authors:** Shaoqi Qu, Shuo Yang, Qingjun Xu, Mengying Zhang, Feng Gao, Yongning Wu, Lin Li

**Affiliations:** ^1^ Animal‐Derived Food Safety Innovation Team College of Veterinary Medicine Anhui Agricultural University Hefei 230036 China; ^2^ Research Unit of Food Safety Chinese Academy of Medical Sciences (No. 2019RU014) NHC Key Laboratory of Food Safety Risk Assessment China National Center for Food Safety Risk Assessment (CFSA) Beijing 100022 China

**Keywords:** bacteria, milk extracellular vesicles, multidrug resistance, plumbagin, polymyxin

## Abstract

The increasing occurrence of infections caused by multidrug‐resistant (MDR) bacteria drives the need for new antibacterial drugs. Due to the current lack of antibiotic discovery and development, new strategies to fight MDR bacteria are urgently needed. Efforts to develop new antibiotic adjuvants to increase the effectiveness of existing antibiotics and design delivery systems are essential to address this issue. Here, a bioinspired delivery system equipped with combination therapy and paracellular transport is shown to enhance the efficacy against bacterial infections by improving oral delivery. A screening platform is established using an in vitro‐induced high polymyxin‐resistant strain to acquire plumbagin, which enhances the efficacy of polymyxin. Functionalized milk extracellular vesicles (FMEVs) coloaded with polymyxin and plumbagin cleared 99% of the bacteria within 4 h. Mechanistic studies revealed that the drug combination damaged the membrane, disrupted energy metabolism, and accelerated bacterial death. Finally, FMEVs are efficiently transported transcellularly through the citric acid‐mediated reversible opening of the tight junctions and showed high efficacy against an MDR *Escherichia coli*‐associated peritonitis–sepsis model in mice. These findings provide a potential therapeutic strategy to improve the efficacy of combination therapy by enhancing oral delivery using a biomimetic delivery platform.

## Graphical abstract

Treating multidrug‐resistant bacteria with a single antibiotic is becoming increasingly difficult, leading to a gradual rise in global infections and deaths. Here, a bio‐inspired delivery system equipped with combination therapy and paracellular transport has been proven to deliver and slowly release a combination of drugs through oral administration, improving the effectiveness of treatment for multidrug‐resistant bacterial infections.

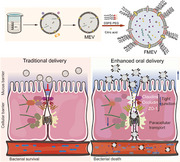

## 1 Introduction

The swift spread of multidrug‐resistant (MDR) bacterial pathogens highlights the need for effective protection against infections.^[^ ^1^ ^]^ In 2019, the Centers for Disease Control and Prevention (CDC) published a report entitled “The Threat of Antibiotic Resistance in the United States,” elucidating the emerging and ongoing trends in bacterial resistance.^[^ ^2^ ^]^ The report focused on 16 antimicrobial‐resistant bacterial pathogens, as well as *Candida infections*, which together account for more than 2 million illnesses and at least 23 000 deaths every year in the United States.^[^ ^3^ ^]^ The CDC highlights that more than half of antimicrobial resistance threats are attributed to gram‐negative bacteria, particularly in certain high‐risk populations.^[^ ^4^ ^]^ The escalation of antibiotic‐resistant infections is driven by several factors, including the overuse and misuse of antibiotics, as well as the lack of development of new antibiotics. Therefore, the unnecessary use of antibiotics needs to be reduced, and new antibiotics and strategies need to be developed to circumvent MDR infections.

The rapid emergence of MDR gram‐negative bacteria, coupled with the sluggish pace of developing new antimicrobial agents, has pivoted our strategy toward integrating conventional antibiotics with nonantibiotic chemosensitizers as a viable approach.^[^ ^5^, ^6^, ^7^, ^8^ ^]^ The combination of chemosensitizers and antibiotics is a typical clinical strategy for ensuring the coverage of potential bacterial pathogens during empirical therapy and for averting dose‐dependent side effects. Compared with synthetic compounds, some active traditional Chinese medicine (TCM) monomers have the benefits of lower toxicity, lower cost, and lower development of resistance; thus, they create a candidate library for use as antibiotic sensitizers.^[^ ^9^, ^10^ ^]^ Medicinal plants are enormous reservoirs of secondary metabolites (PSMs), in which different chemical constituents provide biological activities that can exhibit great pharmaceutical potential.^[^ ^11^, ^12^ ^]^ The antimicrobial properties of PSMs are attributed to their ability to damage bacterial cell membranes, inhibit adenosine triphosphate (ATP) production, interfere with ion transport, and kill bacteria.^[^ ^13^, ^14^, ^15^ ^]^ An increasing number of PSMs have been developed and applied for bacterial infection treatment.^[^ ^16^, ^17^ ^]^ For example, studies have highlighted the potential of plant‐based natural flavonoids, particularly α‐mangostin and isobavachalcone, in combating MDR bacterial pathogens by targeting bacterial membranes and disrupting metabolic functions.^[^ ^18^ ^]^ Therefore, a deep understanding of the potential mechanisms of TCM can contribute to enhancing combination therapy against MDR bacterial pathogens.

The successful codelivery of antibiotics and adjuvants requires appropriate carriers for optimal therapeutic outcomes.^[^ ^19^, ^20^ ^]^ Nanoparticles play an important role in the control of bacterial infections because of their unique physicochemical properties.^[^ ^21^ ^]^ These properties include ultrasmall size, high surface reactivity, large specific surface area, and high drug‐loading capabilities. Moreover, due to these characteristics, they can interact with biological systems at the molecular level, increasing their therapeutic potential.^[^ ^22^, ^23^ ^]^ Furthermore, these properties enable improved drug delivery efficiency, which is critical for the successful eradication of bacterial infections.^[^ ^24^, ^25^, ^26^ ^]^ For example, surface modifications or optimized particle sizes are key factors in the development of effective delivery systems for the accumulation of intracellular drugs.^[^ ^27^ ^]^ Therefore, encapsulating antibiotics and adjuvants within nanoparticles to reprogram the distribution of compounds and maintain sustained release has evolved into an efficacious strategy.

**Scheme 1:**
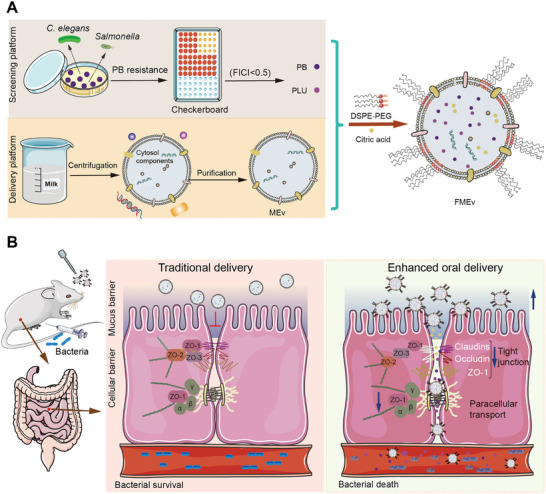
Schematic illustration of the FMEV enhancement in the combination therapy against bacterial infections. A) A screening platform for PB‐resistant *S*. Typhimurium was used to obtain adjuvants for combination therapy. Antibiotics and adjuvants were encapsulated within MEV co‐loaded with citric acid and subsequently functionalized with DSPE‐PEG; this resulted in increased solution stability and mucus penetration. B) Schematic diagram of FMEV with improved oral delivery efficiency. FMEVs were efficiently transcellular transported through the citric acid‐mediated reversible opening of the tight junctions to increase their antibacterial efficacy.

In this work, we introduced a biomimetic delivery system that integrates combination therapy with paracellular transport mechanisms and assessed its antibacterial capability and therapeutic effectiveness in a mouse peritonitis‐sepsis model (**Scheme** **1**). We derived a combination therapy consisting of plumbagin (PLU) and polymyxin (PB) through a screening platform that utilized an in vitro PB‐resistant bacterial strain. Milk extracellular vesicles (MEVs) were functionalized with combination therapy and citric acid (FMEVs) to enhance antibacterial performance and ensure efficient transcellular transport. These results provide a new intervention strategy for enhanced combination therapy with increasing stability and facilitating oral delivery to combat system infections in clinical settings.

## 2 Results

### 2.1 Primary Screening Identified the PLU as an Adjuvant to PB

**Figure 1:**
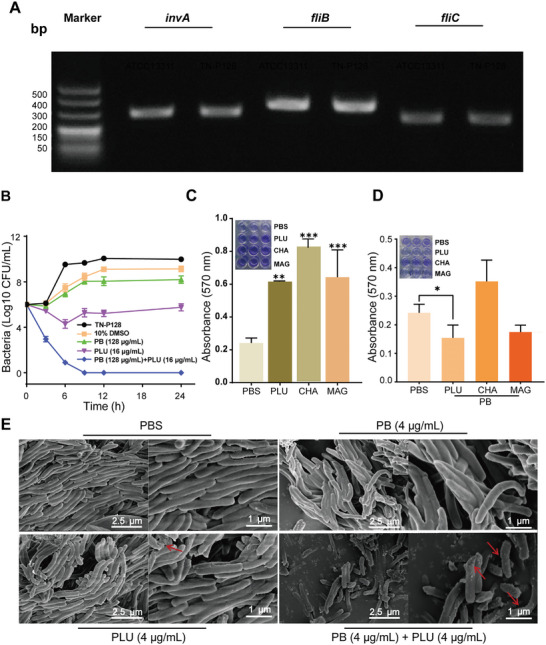
Identification and screening of highly drug‐resistant *S*. Typhimurium. A) PCR identification of TN‐P128. *Salmonella*‐specific genes of *invA*, *fliB*, and *fliC* in both *S*. Typhimurium ATCC13311 and TN‐P128. B) Time‒kill kinetics of TN‐P128 at the exponential growth phase after treatment with PB and PLU. Combining PLU and PB at a 1 × MIC dosage eradicated all bacteria within 6 h, demonstrating a significant synergistic effect. C) Representative images and quantitative analysis of the TNP128 biofilms treated with different drugs. PLU, CHA (chalcone), and MAG (magnolol) alone did not inhibit the generation of biofilms. D) Representative images and quantitative analysis of the TNP128 biofilms treated with different combination therapies. The drug combination of PLU and PB at 1 × FIC significantly reduced the generation of the TN‐P128 biofilms. E) Representative SEM images of TN‐P128 treated with PLU (4 µg mL^−1^), PB (4 µg mL^−1^), or both together. Scale bars represent 2.5 and 1 µm. The SEM images are representative of three independent experiments. Polymyxin B (PB), chalcone (CHA), magnolol (MAG). Data presented as mean ± s.d, *n* = 3. Significance was determined by Tukey's multiple comparisons test following one‐way ANOVA. ns, not significant. *p < 0.05, **p < 0.01, and ***p < 0.001.

To establish a screening platform for antimicrobial TCM, we used *S*. Typhimurium ATCC13311 to induce PB resistance in *C. elegans*. The resistant strain, named TN‐P128, was stable and highly resistant to PB in lysogeny broth (LB) medium, with its minimum inhibitory concentration (MIC) maintained at 128 µg mL^−1^ after eight generations. *Salmonella*‐specific genes *invA*, *fliC*, and *fljB* were identified via PCR; these results confirmed their successful induction (**Figure** **1**A). Next, we determined the MICs of various antibiotics against TN‐P128. Compared with *S*. Typhimurium ATCC13311, TN‐P128 resulted in a significant increase in PB resistance with the following results: a 4‐fold increase in resistance to tetracycline, doxycycline, and mequindox; an 8‐fold increase in resistance to macrolides; and a 64‐fold increase in resistance to florfenicol (Table S1, Supporting Information). Based on these findings, TN‐P128 is suitable as an in vitro model for screening antimicrobial TCM.

Due to the successful fabrication of the screening platform, we further selected and classified the various traditional Chinese medicines (Table S2, Supporting Information). PLU, capsaicin, magnolol, gallic acid, and resveratrol exhibited potent antimicrobial activity (Table S3, Supporting Information). Furthermore, the combination of PLU and PB had a synergistic effect with a significantly lower drug dosage than the other groups (Tables S4, S5, Supporting Information). Importantly, the synergistic effect of PLU and PB resulted in more rapid bactericidal kinetics than the independent use of antibiotics (Figure 1B); these results indicated the robust antibacterial activity of the combination.

Interestingly, previous research has shown that biofilm formation can significantly increase bacterial drug resistance and decrease antibacterial efficacy.^[^ ^28^, ^29^, ^30^ ^]^ Extending these observations, our experiments revealed that the use of TCM alone increased biofilm generation (Figure 1C). Further investigation revealed that the use of TCM alone could inadvertently increase the optical density due to its high concentration and the subsequent substantial drug precipitation. This phenomenon likely accounted for the observed increase in light absorption values. Notably, the combination of PB and PLU significantly reduced biofilm formation (Figure 1D) and accelerated bacterial eradication (Figure S1, Supporting Information). Finally, to observe the morphological alterations in the bacteria after treatment, scanning electron microscopy (SEM) was used to analyze TNP‐128. The cells treated with the PLU and PB dispersions retained a rod‐like structure and were characterized by a smooth exterior and a full‐bodied appearance. Conversely, cells subjected to the PLU and PB mixture presented signs of erosion and slight collapse of the cell wall (Figure 1E; Figure S2, Supporting Information). Overall, the combination of PLU and PB not only had a strong bactericidal effect but could also decrease the formation of bacterial biofilms.

### 2.2 FMEVs Exhibit High Stability and Sustained Release

**Figure 2:**
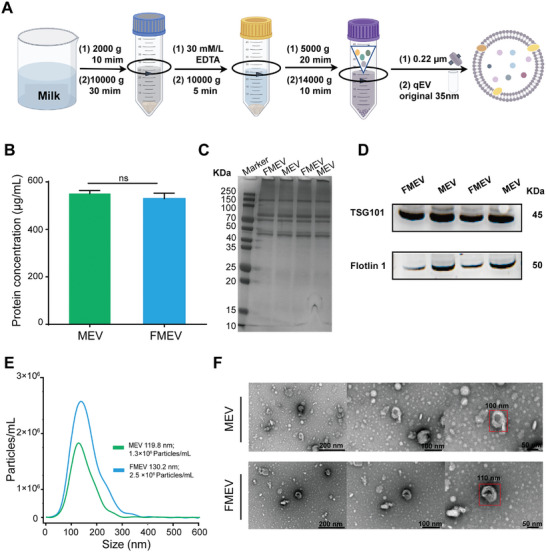
Isolation and characterization of MEV and FMEV. A) Schematic of the isolation process for MEV. B) The protein concentrations of MEV and FMEV were determined via the bicinchoninic acid (BCA) assay. Cholesterol and DSPE‐PEG modifications had no significant effect on the MEV protein concentration. C) The relative abundance of key proteins in MEV was determined by separating the proteins using SDS‒PAGE analysis. MEV and FMEV showed similar distributions of their protein bands. D) Western blotting analysis of the MEV and FMEV. FMEV contained exosome markers, including the membrane protein flotillin‐1 and the ESCRT protein TSG‐101. E) Nanoparticle tracking analysis of MEV and FMEV. Compared to that of MEV, the particle size of FMEV increased. F) Representative transmission electron microscopy images of MEV and FMEV. Data presented as mean ± s.d, *n* = 3. Significance was determined by the unpaired student's *t*‐test. ns, not significant.

To increase the purity of MEV, we developed a method to effectively remove its impurities. The process involves combining EDTA precipitation, ultrafiltration centrifugation, and size exclusion techniques to efficiently separate the MEV (**Figure** **2**A). To maximize the MEV concentration, we used fresh milk from a dairy factory. The efficiency of the MEV isolation from milk was ≈600 µg mL^−1^; this value was much greater than the extraction efficiency of milk powder and related milk products.^[^ ^28^, ^31^ ^]^ The stability of nanoparticles is crucial for sustained release, which can increase their bioavailability. We subsequently modified MEV with DSPE‐PEG to increase its stability. The concentrations of MEVs and FMEVs before and after modification were 530 ±15 µg mL^−1^ and 551 ± 9 µg mL^−1^, respectively (Figure 2B); these results indicated structural integrity after modification.

To confirm the presence of MEV biomarkers, Western blotting was performed on the isolated samples. We observed a similar distribution of the protein bands in MEV and FMEV based on sodium dodecyl sulfate‐polyacrylamide gel electrophoresis (SDS‐PAGE) analysis (Figure 2C). Both the original and modified MEV contained common EV protein biomarkers, such as the protein related to the endosomal sorting complex required for transport (ESCRT) TSG‐101 and the exosome‐associated membrane protein flotillin‐1 (Figure 2D). Additionally, the particle size distribution of the isolated MEV was uniform, with an appropriate median particle size of 119 nm, as determined via nanoparticle tracking analysis. The increased surface zeta potential and particle size of FMEV after the DSPE‐PEG coating process indicated phospholipid encapsulation (Figure 2E; Figure S3, Supporting Information). This appropriate particle size and zeta potential are key factors since they influence the structural stability and drug release.^[^ ^32^ ^]^ Furthermore, transmission electron microscopy (TEM) revealed that the isolated MEV, which was spherical with a lipid bilayer, was crucial for drug loading (Figure 2F). Overall, the large amount of MEVs provided potential vehicles for drug delivery to increase antibacterial efficiency.^[^ ^29^ ^]^

**Figure 3:**
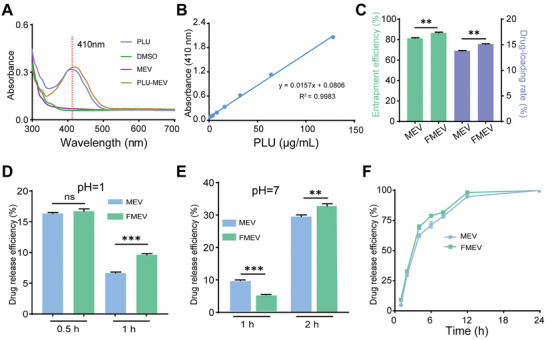
Characterization of the drug loading of FMEV. A) UV spectra of PLU and its solvent DMSO; here, PLU showed maximum uptake at a wavelength of 410 nm. B) Linear regression equation for the PLU concentrations ranging from 0.5 to 128 µg mL^−1^ using a UV spectrophotometer. C) Effects of the DSPE modification on the MEV encapsulation rate and drug loading. D) Drug release rates at pH 1; here, the drug release rates of MEV and FMEV were determined at 0.5 and 1 h. No significant change in the drug release rate was detected within 0.5 h. E) Drug release rates at pH 7; here, the drug release rates of MEV and FMEV were measured at 1 and 2 h. Compared to MEV, FMEV significantly enhanced the drug release rate after 2 h. F) In vitro release profiles of MEV and FMEV at pH 7. All of the above drug release experiments were performed at 37 °C; Data presented as mean ± s.d, *n* = 3. Significance was determined by the unpaired student's *t*‐test. ns, not significant. **p < 0.01 and ***p < 0.001.

To obtain a better understanding of the drug loading rate and entrapment efficiency of MEV, we investigated the concentration of PLU using spectrophotometry. We found that the maximum absorption peak of PLU occurred at 410 nm (**Figure** **3**A). This peak remained unaffected by solvents and MEV, indicating its suitability for quantitative measurements. Moreover, we found that the concentration of PLU in the range of 0.5–128 µg mL^−1^ was linearly related to the absorbance (Figure 3B). To easily obtain PLU‐loaded MEVs, we loaded PLU and PB into the vesicles via a coincubation method. For PLU, when the unloaded drug concentration was determined by spectrophotometry results, the entrapment efficiency and drug loading rate were ≈86% and 16%, respectively (Figure 3C). To explore the stability of MEV modification, we assessed the drug release rate of PLU in MEV and FMEV at different pH values. The results revealed no significant difference in the drug release rate between FMEV and MEV under acidic conditions at 0.5 h; however, the drug release rate of FMEV increased substantially after 1 h (Figure 3D). Under neutral conditions, the drug release rate of MEV exceeded that of FMEV within 1 h, but the rate of FMEV gradually increased and surpassed that of MEV after 1–2 h (Figure 3E,F). These results indicated that FMEVs have desirable environmental stability without affecting drug release efficiency. Furthermore, MEV and FMEV had a certain sustained release effect and played important roles in blood circulation (Figure 3F). Similarly, PB had an entrapment efficiency of 40.8% and a drug loading rate of 5.7% in FMEV (Figures S4A,B, Supporting Information); additionally, PB exhibited rapid initial release in the first 1–4 h and reached a final release rate of 52% after 24 h (Figure S4C, Supporting Information). Initial and loading concentrations of drugs are shown in Table S6 (Supporting Information). Overall, MEVs can be used to facilitate further exploration in drug delivery in vivo.

### 2.3 FMEVs Exhibit High Efficacy in Combating Bacterial Pathogens

**Figure 4:**
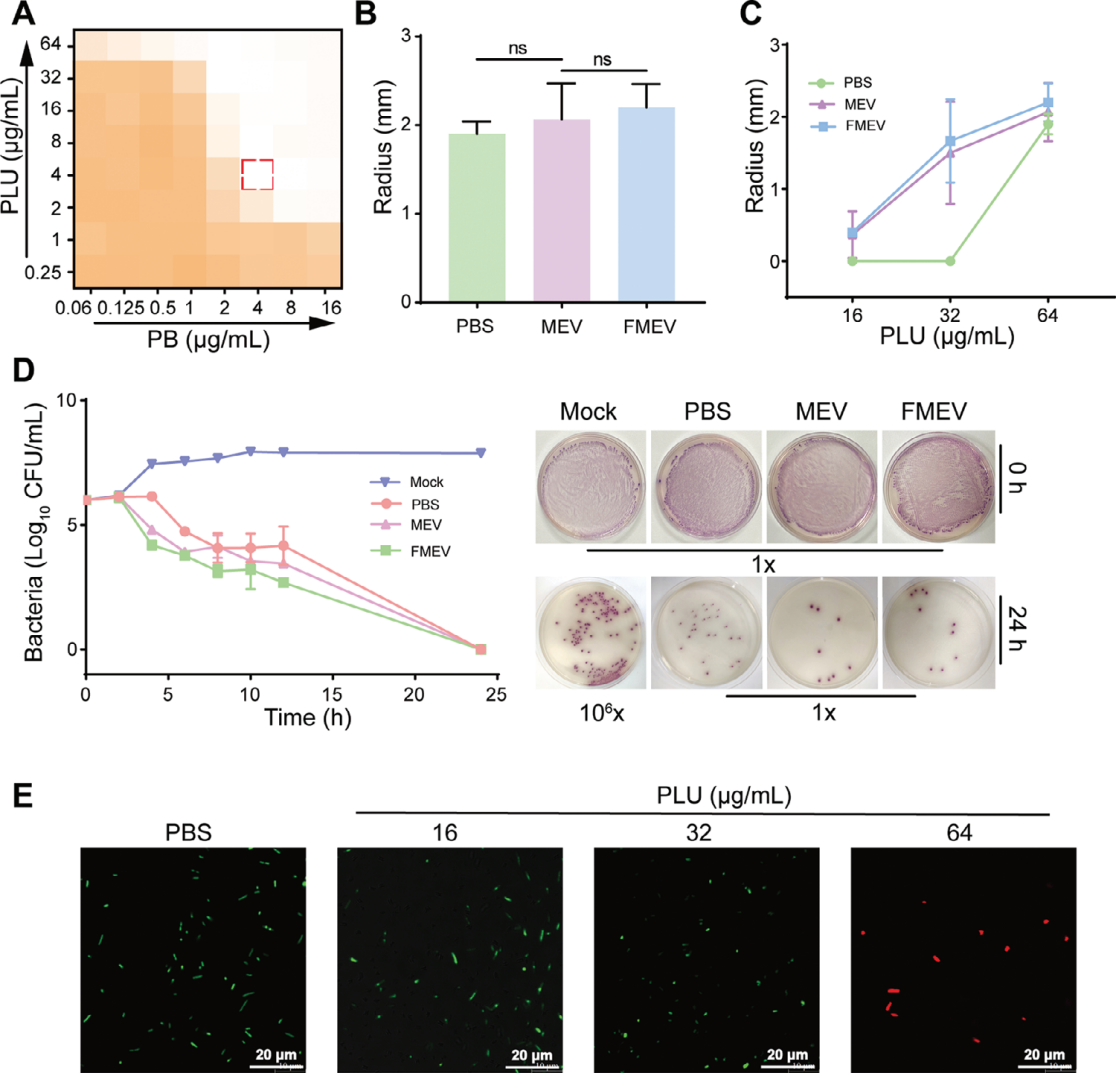
Antibacterial effects of MEV and FMEV on TN‐P128. A) Heatmap showing the drug sensitivity of PB combined with PLU against TN‐P128. B) Inhibition zones for MEV and FMEV (equivalent to the combination of PB with PLU at a concentration of 8 µg mL^−1^ + 64 µg mL^−1^) against TN‐P 128 after overnight incubation at 37 °C. C) The size of the inhibition zones for MEV and FMEV expanded as the PLU concentration increased, particularly in the presence of PB (8 µg mL^−1^). D) Time‒kill kinetics of TN‐P128 at the exponential phase after treatment with FMEV (equivalent to the combination of PB with PLU at a concentration of 8 µg mL^−1^ + 64 µg mL^−1^). E) Representative confocal images of TN‐P128 after treatment with PLU (16–64 µg mL^−1^) in the presence of PB (8 µg mL^−1^) for 1 h. Viable bacterial cells were stained green by SYTO9 (10 µmol L^−1^), whereas dead cells were stained red by PI (10 µmol L^−1^). Data presented as mean ± s.d, *n* = 3. Significance was determined by Tukey's multiple comparisons test following one‐way ANOVA. ns, not significant.

To demonstrate the proof of concept surrounding the codelivery of drug combinations, we measured the antibacterial efficiency against gram‐negative bacterial pathogens via a checkerboard assay and an inhibition zone. The results showed that the combination of PB with PLU had good synergistic antimicrobial activity on TN‐P128 (FICI < 0.5, **Figure** **4**A and Table S3, Supporting Information), and the bactericidal efficiency significantly increased with increasing PLU concentration; these results indicated the concentration‐dependent bactericidal activity of the combination treatment against TN‐P128. Additionally, the disk diffusion assay consistently verified that the high concentration of the combination treatment was bactericidal against TN‐P128 (Figure 4B,C). However, the release of the compounds from MEV onto solid agar plates in the disk diffusion assay could be limited and potentially restrict the observable antimicrobial effects. Furthermore, we observed that FMEV eradicated 99.9% of TN‐P128 after incubation for 8 h (Figure 4D; Figure S5, Supporting Information). Moreover, the dose‐dependent bactericidal activity was further confirmed by the increased numbers of bacteria ranging from live (green) to dead (red) using a live/dead bacterial viability assay (Figure 4E). These results collectively demonstrate that MEV loaded with a combination of PB and PLU has a formidable synergistic effect on the sterilization of bacterial pathogens.

### 2.4 Antibacterial Mechanisms of PLU Combined with PB

**Figure 5:**
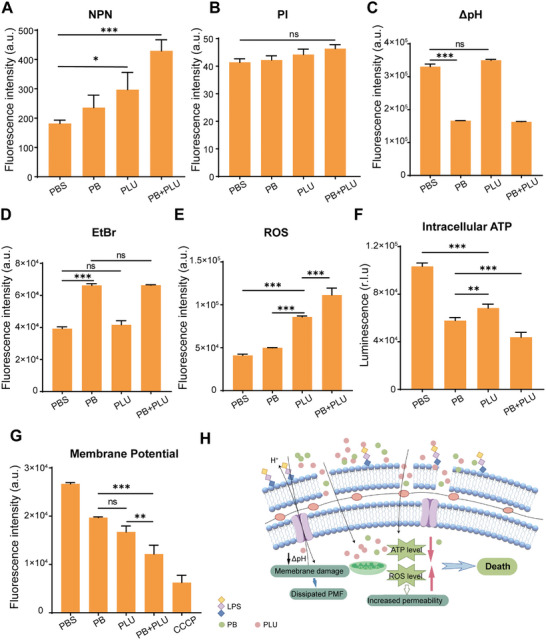
Mechanism of PLU in combination with PB against TN‐P128. A) Synergistic treatment with PB and PLU at 1/2 × FIC compromised the outer membrane integrity of TN‐P128, as shown by the NPN uptake. B) Inner membrane integrity of TN‐P128 was maintained after the combined PB and PLU treatment, with the 1/2 × FIC of PLU and PB showing no significant impact when assessed with the PI. C) Dissipation of the ΔpH by PB in TN‐P128. Exponential TN‐P128 was incubated with the pH fluorescence probe BCECF‐AM. Cotreatment with PLU and PB at 1/2 × FIC had no synergistic effect. D) TN‐P128 results after PB treatment. After PB treatment, TN‐P128 resulted in reduced efflux pump levels, and the combined use of PLU and PB at 1/2 × FIC demonstrated no cooperativity. E) PLU treatment enhancement of the ROS accumulation in TN‐P128 cells, as measured with the fluorescence probe DCFH‐DA (10 µmol L^−1^). The combined PLU and PB treatment at 1/2 × FIC significantly synergized the increase in intracellular ROS. F) Combination of PLU and PB results. The combination of PLU and PB significantly reduced the intracellular ATP levels in the TN‐P128 cells, demonstrating a synergistic effect at a concentration of 1/2 × FIC, as measured by a luminescent ATP assay. G) Induced membrane depolarization in TN‐P128 by both PB and PLU treatments; a synergistic effect was observed with 1/2 × FIC, as shown by DiOC2(3) staining. H) Schematic diagram of the mechanism of action of the combination therapy in gram‐negative bacteria. Data presented as mean ± s.d, *n* = 3. Significance was determined by Tukey's multiple comparisons test following one‐way ANOVA. ns, not significant. *p < 0.05, **p < 0.01, and ***p < 0.001.

The structural integrity and functional capacities of the bacterial membranes are critical for the growth and survival of bacteria, particularly concerning the inner and outer membranes of gram‐negative bacteria.^[^ ^33^ ^]^ Studies have demonstrated that the primary target of PB is the lipopolysaccharide present in the outer membrane of gram‐negative bacteria.^[^ ^34^ ^]^ To analyze the synergistic mechanism of PB and PLU based on previous experimental methods,^[^ ^18^ ^]^ we used the highly drug‐resistant *S*. Typhimurium TN‐P128 as a model. First, we determined the integrity and fluidity of the bacterial membrane using the NPN (N‐Phenyl‐1‐naphthylamine), TMA‐DPH (N,N,N‐trimethyl‐4‐(6‐phenyl‐1,3,5‐hexatrien‐1‐yl), and PI (propidium iodine) probes. We found that the integrity of the bacterial outer membrane was damaged after the combination therapy treatment, whereas the bacterial inner membrane remained intact (**Figure** **5**A,B). This finding was consistent with the membrane fluidity results showing that PLU and PB combined in the combination therapy caused a greater decrease in the fluidity than either agent alone (Figure S6A, Supporting Information). Moreover, we monitored protein and nucleic acid leakage after antibiotic treatment for 24 h. The results revealed that neither single nor combined drug application significantly affected the bacterial protein and nucleic acid leakage (Figure S6B,C, Supporting Information). These results were consistent with the changes in the integrity of the outer membrane; these changes disrupted the bacterial homeostasis and led to the dissipation of basic metabolic disorders, including proton dynamics. Consequently, we used the fluorescent probe BCECF‐AM (2′,7′‐bis‐(2‐carboxyethyl)−5‐(and‐6)‐carboxyfluorescein, acetoxymethyl ester) to evaluate the change in pH (ΔpH), which is the key component of proton dynamics in bacteria.^[^ ^35^ ^]^ Compared with PB, PLU alone did not significantly reduce ΔpH in a short time (Figure 5C). To further verify the reason for the bacterial ΔpH, we determined the level of the bacterial proton pump using the fluorescent dye ethyl bromide (EtBr) (Figure 5D). The results were in agreement with the ΔpH observations and were primarily attributed to the substantial impact of the proton pump on PB.

Next, to investigate whether oxidative stress in bacteria was triggered by superoxide or reactive oxygen species, we conducted a reactive oxygen species (ROS) assay to monitor the oxidative stress levels. The results revealed that PLU significantly elevated the bacterial oxidative stress, and this increase was more intense using the combination therapy (Figure 5E). We subsequently assessed bacterial ATP (adenosine 5′‐triphosphate) changes and the membrane potential using an ATP assay kit and the fluorescent dye DiOC(2)3 (3,3′‐diethyloxacarbocyanine iodide). Here, the combination of PB and PLU significantly reduced the intracellular ATP content, while the extracellular ATP content did not significantly change (Figure 5F; Figure S6D, Supporting Information); these results indicated the inhibition of bacterial ATP production and transport. These observations were further corroborated by the alterations in the bacterial membrane potential induced by PB and PLU (Figure 5G), and the combination therapy significantly enhanced these effects. We detected an increase in the accumulation of PLU in the bacterial cytoplasm (Figure S6E, Supporting Information), which was likely attributed to the binding of PB to outer membrane lipid A. At the same time, the increased ROS level intensified the oxidative stress and accelerated the bacterial death (Figure 5H). Overall, the results indicated that the membrane depolarization induced by both PLU and PB compromised the integrity of the outer membrane, leading to membrane dysfunction and increased antibacterial efficacy.

### 2.5 FMEV Shows High Efficacy in a Mouse Peritonitis‐Sepsis Model

MEVs possess remarkable biocompatibility because of their chemical composition, low immunogenicity, and non‐cytotoxicity.^[^ ^28^ ^]^ The hydrophobic and hydrophilic bioactive molecules delivered by MEVs show excellent stability in the digestive tract; thus, they are suitable candidates for the development of an oral drug delivery system.^[^ ^29^ ^]^ To ensure the feasibility of in vivo application, a safety evaluation was performed before the pharmacodynamic assay. We observed an absence of hemolysis in PLU across concentrations ranging from 8 to 256 µg mL^−1^, as well as in MEVs and FMEVs based on the in vitro hemolysis assay (Figure S7A,B, Supporting Information). Furthermore, the mice were orally administered the FMEVs to assess biochemical indices post‐intestinal absorption. The results confirmed that the critical indices, including phosphorus (P), calcium (Ca), glucose (Glu), alkaline phosphatase (ALP), total cholesterol (TC), and albumin (ALB) levels, remained stable and within healthy limits (Figure S7C, Supporting Information). Moreover, in vitro exposure of RAW 264.7 and HepG2 cells to FMEVs (at concentrations of 1, 5, 10, 20, 50, and 100 µg mL^−1^) for 24 h confirmed its nontoxic nature to both cell types (Figure S8, Supporting Information). In summary, these findings indicate that FMEVs have potential in medical and pharmaceutical applications.

**Figure 6:**
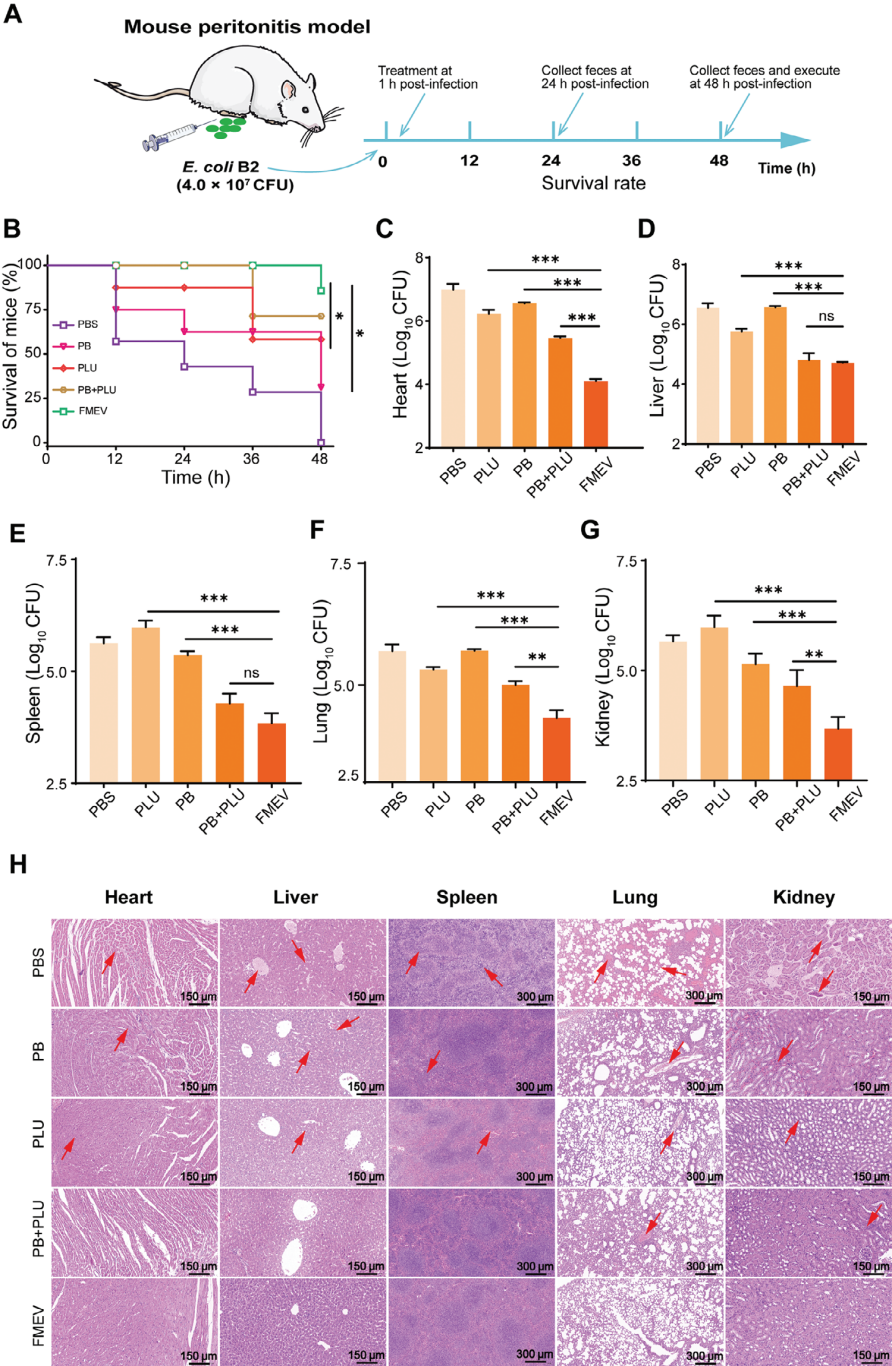
Improved of the therapeutic effect in a peritonitis–sepsis infection model in mice by using FMEV. A) Scheme of the experimental protocol for the mouse peritonitis–sepsis model. B) Survival rates of mice with peritonitis–sepsis (*n* = 7). Mice were infected with a lethal dose of *E. coli* B2 (4.0 × 10^7^ CFU) in the presence of PBS, PB (8 mg kg^−1^), PLU (16 mg kg^−1^), PB + PLU (8 mg kg^−1^ + 16 mg kg^−1^), and FMEV (PB: 8 mg kg^−1^ + PLU: 16 mg kg^−1^). *P*‐values were determined using the two‐sided, log[rank] (Mantel–Cox) test. *p < 0.05. C–G) Bacterial load (expressed as Log10 CFU) of *E. coli* B2 present in the heart (C), liver (D), spleen (E), lung (F), and kidney (G); this was evaluated at 48 h via a tenfold serial dilution technique. H) Representative photomicrographs of the H&E and Masson staining of the heart, liver, spleen, lung, and kidney. The arrow indicates the pathological lesions. The results are presented as the mean ± s.d. (*n* = 7). Significance was determined by Tukey's multiple comparisons test following one‐way ANOVA. ns, not significant. **p < 0.01 and ***p < 0.001.

Due to the attractive biological activity and in vivo safety of FMEVs in vitro, we investigated its therapeutic potential in a peritonitis‐sepsis model in mice infected with *E. coli* B2 and TN‐P128 (**Figure** **6**A). We found that TN‐P128 (1.0 × 10^9^ CFU) failed to kill more than half of the mice within 48 h (Figure S9, Supporting Information). Then, the mice were intraperitoneally infected with a dose of 4.0 × 10^7^ CFU of *E. coli* B2 suspension for 1 h before being treated with a 200 µL mixture of FMEVs and citric acid (4 g L^−1^), according to a previous method with slight modifications.^[^ ^36^ ^]^ Only 28% of the mice in the control group after treatment with PB for 48 h; however, more than 85% of the mice in the control group survived after a single dose of FMEVs (Figure 6B). Since PLU had a potent antibacterial effect, most mice (56%) survived after treatment with a single dose of PLU (16 mg kg^−1^) within 48 h. Furthermore, FMEVs significantly reduced the bacterial load in major organs, including the heart, liver, spleen, lung, and kidney, by more than 99%; these results indicated robust antibacterial activity (Figure 6C–G). No evident inflammation and atrophy were detected in the cecum of the mice, and the fecal bacterial loads were significantly lower in the FMEV‐treated mice than in the PLU‐ or PB‐treated mice (Figure S10A,B, Supporting Information). Moreover, this activity was further confirmed by analyzing tissue sections stained with hematoxylin and eosin (H&E). We found that the pathological changes and tissue damage in multiple organs were decreased by FMEV therapy (Figure 6H). Based on these results, a single FMEV mixed with citric acid is effective in eliminating bacteria and improving survival.

**Figure 7:**
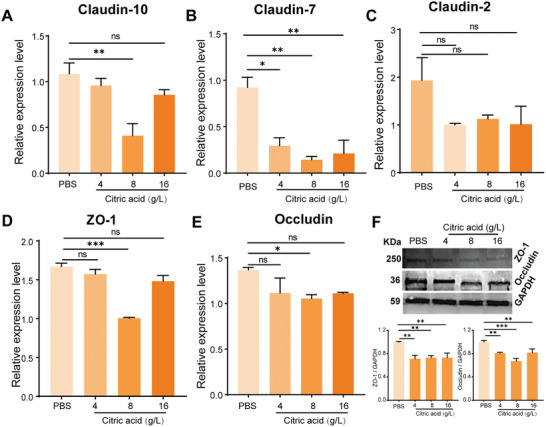
Mechanism of citric acid‐mediated reversible opening of the tight junctions to increase transcellular transport. A–E) Relative expression and quantification of the tight junction‐related protein genes Claudin‐10 (A), Claudin‐7 (B), Claudin‐2 (C), ZO‐1 (D), and Occludin (E) in the intestines of mice treated with 200 µL of citric acid at concentrations of 4, 8, and 16 g L^−1^. The expression of genes related to the tight junction was decreased. F) Quantitative analysis of the expression of the tight junction proteins ZO‐1 and Occludin in the intestines of mice treated with citric acid, as determined by Western blotting. Data presented as mean ± s.d, *n* = 3. Significance was determined by Tukey's multiple comparisons test following one‐way ANOVA. ns, not significant. *p < 0.05, **p < 0.01, and ***p.

To further explore the mechanism of paracellular transport of FMEV in vivo, we performed gene and protein expression tests using real‐time quantitative polymerase chain reaction and Western blotting. Citric acid could open tight junctions in mouse intestines, thereby enhancing drug penetration and absorption to a certain extent.^[^ ^37^ ^]^ Mice were intragastrically administered a range of 4–16 g L^−1^ citric acid solution (200 µL), and the duodenum and jejunum were collected for further determination. The gene levels of Claudin‐10, Claudin‐7, and Claudin‐7 were strongly decreased in response to FMEV treatment (**Figure** **7**A–C); these results were in agreement with the increased paracellular transport through opening tight junctions. Furthermore, the gene levels of ZO‐1, ZO, and Occludin in the jejunum were significantly decreased under FMEV treatment (Figure 7D,E; Figure S11, Supporting Information). Similarly, the protein expression of ZO‐1 and Occludin was significantly lower than that in untreated mice, which contributed to the opening of the tight junctions (Figure 7F). Similarly, citric acid treatment significantly reduced the protein expression of ZO‐1 and Occludin in ICE‐6 cells in a concentration‐dependent manner (Figure S12, Supporting Information). Nanoparticles were efficiently transported across cells via paracellular transport to increase oral bioavailability; this involved the temporary opening of tight junctions.^[^ ^38^ ^]^ Based on these results, the potential of FMEV as a platform to enhance efficacy was demonstrated using an effective oral delivery approach.

## 3 Discussion

The misuse and overuse of antibiotics have increased the prevalence of MDR bacterial pathogens.^[^ ^39^, ^40^ ^]^ Due to increased tolerance and low absorption efficiency during treatment, the effectiveness of traditional antibiotics is severely limited. In this study, PLU was chosen from a variety of TCM as the chemical sensitizer for PB based on an in vitro screening platform using induced PB‐resistant *S*. Typhimurium as a model. PLU and PB coloaded MEV were functionalized with DSPE‐PEG and citric acid, which is a type of intestinal permeability enhancer,^[^ ^41^ ^]^ to enhance gastrointestinal stability and paracellular transport. This multifunctional platform elucidates alternative strategies to increase antibacterial efficacy by facilitating oral delivery.

Oral drug delivery is often preferred for treatment because of the complexities and expenses associated with intravenous administration.^[^ ^42^ ^]^ It provides advantages such as being painless, convenient, and easily self‐administered. However, certain drugs, such as peptides and proteins, may require injection because of their poor oral bioavailability; this results from low peptidase tolerance and intestinal permeability.^[^ ^43^ ^]^ Although capsule preparations have overcome many of the harsh conditions of the gastrointestinal tract, oral peptide preparations still need to be taken before meals to prevent premature release during the food buffering process. This aspect highlights the importance of designing formulations for controlled, sustained, and targeted drug delivery to increase drug efficacy. Among the various oral administration methods, mucosal adhesion carrier emerges as being particularly advantageous.^[^ ^44^ ^]^ It enhances the retention and controlled absorption of oral drugs, potentially treating both local diseases in the gastrointestinal tract and systemic diseases.

Notably, milk contains many biocompatible EVs that can maintain the integrity of phospholipid vesicles for extended periods in vitro.^[^ ^45^ ^]^ Further surface modifications can improve adhesion and biocompatibility.^[^ ^46^ ^]^ The inefficiency of oral delivery is not only due to misuse but is also caused by low drug solubility. Therefore, effective oral administration of antibacterial drugs, especially Biopharmaceutics Classification System II and IV compounds with low water solubility, is the best treatment approach.^[^ ^47^ ^]^ Biological or biomimetic drug carriers, including EVs, have successfully enhanced therapeutic effects by improving their bioavailability.^[^ ^48^ ^]^ However, Due to their low yield and high cost, EVs derived from the cell lines and bacteria are impractical for commercial drug production. Milk, which is a rich source of EVs, provides a promising alternative despite the complexity of extraction due to the presence of non‐EV proteins, sugars, milk fat, and other components. Importantly, MEVs exhibit remarkable resilience, surviving strong acidity in the stomach and degradation in the intestines while crossing biological barriers to reach target tissues.^[^ ^49^ ^]^ Due to this ability to traverse the gastrointestinal barrier, MEVs are promising vehicles for oral drug delivery.

Drug absorption in the gastrointestinal tract can be categorized into two pathways: transcellular and intracellular.^[^ ^50^ ^]^ The effectiveness of these pathways can be increased by a mucosal adhesive carrier delivery system.^[^ ^51^ ^]^ Paracellular absorption, which is part of the transcellular pathway, is often restricted by tight junctions and paracellular pathways.^[^ ^52^ ^]^ These junctions are composed of transmembrane integrins (claudins), junction adhesion molecules, plaque proteins, and regulatory proteins.^[^ ^53^, ^54^ ^]^ The absorption capacity of mucosal adhesion carriers is influenced by factors such as their size, material, and shape.^[^ ^55^ ^]^ Additionally, during intestinal absorption, the absorption of these carriers is affected by the paracellular absorption.^[^ ^56^, ^57^ ^]^ Theoretically, enhancing paracellular uptake can improve the absorption and penetration efficiency of drugs. In the past, intestinal permeability enhancers were used to improve the oral delivery of therapeutic peptides, but their impact on tight junction disruption and paracellular absorption was disregarded.^[^ ^41^ ^]^ For example, citric acid is an intestinal permeability booster, and a small amount of citric acid strengthens the intestinal tight junction barrier;^[^ ^58^ ^]^ however, at an increased dose, it mainly alters the intestinal permeability, which enhances the cellular paracellular absorption and paracellular osmosis.^[^ ^59^ ^]^ Therefore, we chose citric acid as an intestinal permeability enhancer. This enhancer can aid in the opening of the tight junctions of the intestinal epithelium and increase the absorption and utilization of drugs through intragastric administration.

In summary, we have clearly shown that the combination of an antibiotic discovery platform with precision delivery greatly benefits the treatment of MDR bacterial infection. A new combination therapy using PLU and PB was developed via a PB‐resistant *S*. Typhimurium screening platform. This combination was encapsulated in MEVs and functionalized with PEG and citric acid for a precise delivery system to facilitate oral delivery. Based on our strategy, as a multifunctional platform, FMEVs could enable the slow release of antibiotics, paracellular transport, and enhanced efficacy.

## 4 Experimental Section

### Materials

PLU, PB, citric acid, resveratrol, and capsaicin were purchased from Maclin Biochemical Technology (Shanghai, China). Tetracycline, gallic acid, tigecycline, and ampicillin were purchased from Solaibao (Beijing, China). Chloramphenicol, artemisinin, chalcone, baicalein, berberine chloride hydrate, puerarin, ursolic acid, matrine, paraformaldehyde, glycerol, and anhydrous sodium sulfate were purchased from Aladdin (Shanghai, China). Spectinomycin hydrochloride, coumarin, baicalin, ciprofloxacin, meropenem, and bacteresulf were purchased from Yuanye Bio‐Technology (Shanghai, China). Phosphatase inhibitor mixture A (50 ×, P1081), ZO‐1 rabbit polyclonal antibody (AF8394), occludin polyclonal antibody (AF7644), live dead bacteria staining kit (EX3000), GAPDH rabbit monoclonal antibody(AF1186), BCA protein concentration assay kit, propidium iodide, enhanced ATP assay kit, reactive oxygen species detection kit, BCECF AM (S1006) were purchased from Biyuntian Biotechnology (Shanghai, China). RNAase‐free in double steam, total RNA extraction reagent, electrophoretic precast adhesive, trizol, and 5 × protein loading dye were purchased from BioEngineering (Shanghai, China). Amicon Ultra‐15 Centrifugal Filter Unit (UFC501024), Amicon Ultra‐0.5 Centrifugal Filter Unit (UFC903024), liposomal extruder and acetonitrile were purchased from Merck (Germany). 1,2‐Distearoyl‐sn‐glycero‐3‐phosphorylethanolamine (DSPE), DSPE‐PEG2000, and cholesterol (CHO) were purchased from AVT Pharmaceutical Technology (Shanghai, China). CAMHB broth (HB6231‐1), BHI broth (HB8297‐1), agar powder (HB8274‐1), *Salmonella* chromogenic medium (HB7007‐1), *E. coli* chromogenic medium (HB7003‐7) were purchased from Haibo (Qingdao, China). All reagents were analytical grade and used without further purification.

### Bacterial Strains and Cultivation

The bacterial strains used in this study are listed in Table S1 (Supporting Information)*. Escherichia coli* (*E. coli*) ATCC 25922 was purchased from the Guangdong Microbial Seed Conservation Center (GDMCC). *Salmonella* Typhimurium (*S*. Typhimurium) ATCC 13311 and *E. coli* B2 were stored in the laboratory. All of the above bacteria were routinely cultured and amplified in Luria‐Bertani (LB, HB0128) medium.

### Screening Platform of PB‐Resistant Strain

*Caenorhabditis elegans* (*C. elegans*) was used as an *S*. Typhimurium ATCC 13311 host. The *C. elegans* were grown in liquid before being parasitized by *S*. Typhimurium. Initially, they were fed PB at a concentration equal to half the MIC of *S*. Typhimurium. The concentration of PB in the culture medium was gradually increased until *S*. Typhimurium showed stable antibiotic resistance. These induced resistant bacteria were then grown in a blank medium for 5–10 generations. Antibiotic susceptibility tests were carried out to ensure stable resistance to PB. The drug‐resistant strain was named TN‐P128. Moreover, primers for PCR identification were designed using Prime 5, based on the sequence of the *Salmonella*‐specific gene (*invA*) and flagella‐related genes (*fliC* and *fljB*). The primer sequence information is obtained in Table S7 (Supporting Information).

### Determination of Antimicrobial Susceptibility

The minimum inhibitory concentration (MIC) of antibiotics was determined using the broth microdilution method, following the 2017 guidelines set by the Clinical Laboratory Standards Institute. Briefly, the antibiotics were diluted in CAMHB broth and combined them with an equal volume of bacterial suspensions. These suspensions had ≈1.5 × 10^6^ colony‐forming units (CFU)/mL. The mixture was then placed into a 96‐well microtitre plate and left it to incubate for 18 h at 37 °C. The MIC was defined as the smallest antibiotic concentration that stopped any visible bacterial growth.

### Checkerboard Assays

The combined effects of TCM and PB were determined using checkerboard assays. In a nutshell, both drugs were diluted two‐fold in the culture medium, mixed them with an equal volume of bacterial suspensions in CAMHB containing ≈1.5 × 10^6^ CFUs mL^−1^, and then placed them in a sterilized 96‐well plate. After 18 h of culture at 37 °C, the MICs were recorded and evaluated the synergistic effect by calculating the Fractional Inhibitory Concentration (FIC). Synergy was defined as an FIC index ≤0.5.

### Biofilm Formation Assays

*S*. Typhimurium at the exponential phase was adjusted to roughly 1.5 × 10^8^ CFU mL^−1^. 200 µL of bacterial solution was added to each well of sterilized 96‐well plates. The control group received an equal amount of LB broth and ddH_2_O. The bacteria were then combined with 1 × MIC and 1 × FIC of drugs and incubated at 37 °C for 48 h. The bacterial solutions were gently disposed and mixed each well with 200 µL of methanol for 15–30 min, followed by three washes with sterile PBS. Once air‐dried, 200 µL of 0.1% crystal violet was added and stirred before washing three times with sterile PBS. After another round of air‐drying, the samples were mixed with 200 µL of acetic acid for 30 min to dissolve any precipitate. Finally, the OD_570_ value of each well was measured using a microplate reader to evaluate the biofilm formation capacity.^[^ ^60^ ^]^

### Observation of Bacterial Cell Membranes by Scanning Electron Microscope

TN‐P128 and *E. coli* B2 were grown overnight and then diluted to a turbidity of 0.5. They were then co‐cultured with the drug for 2 h at 37 °C. Bacteria were treated with PBS, PB (2 µg mL^−1^), PLU (16 µg mL^−1^), and PB + PLU (2 µg mL^−1^ + 16 µg mL^−1^ respectively). After incubation, the samples were centrifuged at 10000 × g for 5 min and then fixed with 2.5% glutaraldehyde at 4 °C. Four hours later, they were placed overnight in a freeze‐drying apparatus (Biocool FD‐1A‐50), and images were captured using a scanning electron microscope (Hitachi S‐4800).

### Isolation of MEV

MEV was extracted from fresh milk bought from Ansong Dairy Family Farm (Anhui, China) through a mix of differential centrifugation and size exclusion chromatography. All steps were performed at 4 °C. Initially, the milk was centrifuged at 2000 × g for 10 min and 10 000 × g for 30 min to collect the supernatant. Ethylene diamine tetraacetic acid (EDTA) was added at 20 mm L^−1^ and collected the supernatant at 10 000 × g for 10 min. 15 mL of the supernatant was added to an Amicon Ultra‐15 centrifuge tube and centrifuged at 5000 × g for 20 min. Then, 0.5 mL of enrichment solution was added to the Amicon Ultra‐2.5 ultrafiltration centrifuge tube and centrifuged at 14 000 × g for 10 min. The resulting liquid was filtered through a 0.22 µm membrane and further purified using Izon qEV original/35 nm columns. MEV were dispersed in PBS and stored at −20 °C until used.

### Modification of MEV

To improve the stability of MEV, exogenous phospholipids were added to adjust lipid composition. DSPE and cholesterol were dissolved in trichloromethane and evaporated to dryness at room temperature overnight. A dried film forming a cloudy, hard gel‐like material at the bottom of the vial was observed. Next, the resulting film was hydrated with MEV and stirred at 60 °C for 20 min. 1 mL of the mixture was extruded using the liposomes extruder (Avanti 610020) with two polycarbonate membranes (200 nm pore sizes, Whatman, USA) and repeated 22 times.

### Characterization of EVs

The identification of EVs refers to the previous exosome identification methods.^[^ ^29^, ^61^ ^]^ First, the BCA protein concentration assay kit was utilized to ascertain the concentrations of MEV proteins. SDS‐PAGE was employed for detecting the total protein content and distribution range in the raw materials. To measure the particle size and concentration of MEV, Nanoparticle tracking analysis (NTA) was performed using Zeta View PMX 110 (Particle Metrix Ltd, Germany) at a dilution of 10^6^. The surface zeta potential of MEV was assayed by dynamic light scattering using a Malvern Zetasizer Nano ZS (U.K.). The ≈1 × 10^5^ particles of MEV and FMEV were examined using a transmission electron microscope (TEM, JEM‐1200, Japan).

### Western Blots

After determining the protein concentration, the protein was boiled for 10 min, and then separated via SDS‐PAGE in 15% gels, followed by transfer to a nitrocellulose membrane. The membranes were primed with methanol for a minute and then immunoblotted with primary antibodies for rabbit anti‐TSG101 antibody (AB125011, Abcam) and rabbit anti‐Flotillin 1 antibody (AB133497, Abcam). After first overnight incubation with the antibody, the PVDF membrane was washed three times with TBST on a decolorization shaker, then incubated with the secondary antibody at 4 °C for an hour, followed by another round of TBST washing. Proteins reacted to the antibodies were then visualized using a gel imaging system.

### Evaluation of Drug Loading

The absorbance of varying drug concentrations at 410 nm was analyzed, followed by a linear regression analysis. This led to the generation of a standard curve equation for PLU concentration within the range of 0.5–128 µg mL^−1^, which was based on concentration and absorbance values. The encapsulation efficiency (EE) and loading efficiency (LE) of FMEV were separately determined via ultrafiltration centrifugation, where the initial mixing concentration of PLU and PB was 90 and 70 µg mL^−1^. For the computation of EE and LE, the following formula was utilized:

(1) $$\mathrm{EE}\;\left(\%\right)=\left(\mathrm{mass}\;\mathrm{of}\;\mathrm{loaded}\;\mathrm{drug}\;/\mathrm{mass}\;\mathrm{of}\;\mathrm{total}\;\mathrm{drug}\right)\times \;100$$

(2) $$\mathrm{LE}\;\left(\%\right)=\left(\mathrm{mass}\;\mathrm{of}\;\mathrm{loaded}\;\mathrm{drug}\;/\;\mathrm{mass}\;\mathrm{of}\;\mathrm{FMEV}\right)\times \;100$$

### In Vitro Drug Release

To test the impact of gastrointestinal pH on the drug release rates of FMEV, the environmental conditions were adjusted using hydrochloric acid. This mimics the unique conditions in the stomach and intestines. The in vitro release rates of FMEV were evaluated at 0.5 and 1 h under the condition of pH 1. Additionally, the release profiles of FMEV were examined for 24 h at pH 7.

### Disk Diffusion Assay

The antibacterial efficiency of FMEV was tested using the disk diffusion assay. LB agar plates were used, inoculated with a TN‐P128 suspension (1 × 10^7^ CFUs) for maximum growth. Inhibitor disks were prepared by combining the PB concentration (8 µg mL^−1^) with high (PLU 64 µg mL^−1^), medium (PLU 32 µg mL^−1^), and low (PLU 16 µg mL^−1^) concentrations. 50 µL of this mixture was then applied to a 6‐mm diameter filter paper. The plates were then placed in a 37 °C environment for 16 h. The diameter of the zone with no growth was measured to identify the inhibition zones. A 6‐mm zone of inhibition indicates no inhibition.

### Bacterial Viability Assay

TN‐P128 bacteria was obtained in the exponential phase and adjusted them to a concentration of 1 × 10^8^ CFU mL^−1^. The concentration of PB was held steady at 8 µg mL^−1^ while combining it with PLU high (64 µg mL^−1^), medium (32 µg mL^−1^), and low (16 µg mL^−1^) concentrations. The bacteria were then exposed to the drug for 1 h. SYTO9 (10 µmol L^−1^) and PI (10 µmol L^−1^) were added to reach a final volume of 1 mL and stored at room temperature for 15 min. Fluorescent images of the stained bacteria were captured using confocal laser‐scanning microscopy (Leica, Germany).

### Analysis of Inner and Outer Membrane Integrity

Inner membrane: TN‐P128 bacteria was obtained in an exponential growth phase, washed them twice with 5 mmol L^−1^ HEPES (supplemented with 5 mmol L^−1^ glucose), and adjusted to OD_600_ = 0.5. Subsequently, PI was added to a final concentration of 15 µmol L^−1^ and incubated at 37 °C for 30 min. The bacterial suspension was divided into four groups: control, PB (2 µg mL^−1^), PLU (2 µg mL^−1^), PB (2 µg mL^−1^) + PLU (2 µg mL^−1^), each with a total volume of 1 mL. After thorough mixing, the samples were incubated at 37 °C for 1 h. The fluorescence intensity was measured using a multifunctional microplate reader with excitation wavelengths of 535 and 615 nm.

### Outer Membrane

TN‐P128 bacteria in the exponential phase were collected and washed twice with 5 mmol L^−1^ HEPES (containing 5 mmol L^−1^ glucose). The bacterial suspension was adjusted to OD_600_ = 0.5 and then 1‐n‐phenyl enaphthalamine (NPN) was added to a final concentration of 10 µmol L^−1^. The mixture was then incubated at 37 °C for 30 min. Antibiotic treatment was the same as above, with a total volume of 1 mL, fully mixed, and incubated at 37 °C for 1 h. Next, the fluorescence intensity was determined using a multifunctional microplate reader at the excitation wavelength of 350 nm and the emission wavelength of 420 nm. All tests were performed in triplicate.

### Determination of Membrane Potential

TN‐P128 bacteria in the exponential phase were collected and adjusted to a turbidity of 0.5 (1 × 10^8^ CFU mL^−1^). The bacterial solution was then diluted with sterile PBS to a bacterial density of ≈1 × 10^7^ CFU mL^−1^ and added to a final concentration of 50 µg mL^−1^ DiOC_2_(3). The mixture was then incubated at room temperature for 20 min. The drug treatment was the same as above and then incubated at 37 °C for 1 h. Finally, fluorescence intensity was determined using a multifunctional microplate reader with excitation at 486 nm and emission at 620 nm. All tests were performed in triplicate.

### Intracellular and Extracellular ATP Determination

TN‐P128 bacteria in the exponential growth phase were collected and adjusted to a turbidity of 0.5 (1 × 10^8^ CFU mL^−1^). The bacteria were then diluted with sterile PBS to a bacterial density of ≈1 × 10^6^ CFU mL^−1^. The drug treatment was the same as above and incubated at 37 °C for 1 h. After incubation, the mixture was centrifuged at 5000 × g for 5 min. The bacteria were gently precipitated and lysated followed by centrifuged at 4 °C and at 12 000 × g for 5 min. Next, 100 µL of the ATP test working solution was added into the black 96‐well plate at room temperature and quickly added 100 µL of the supernatant. The mixture was then placed into the microporous plate‐type luminescence detector (SpectraMax 190) for detection and recorded the data. All tests were conducted in triplicate.

### Reactive Oxygen Species (ROS) Determination

TN‐P128 in the exponential growth phase were collected and washed three times with PBS buffer. The bacteria were then resuspended in the buffer to create a bacterial suspension with OD_600_ = 0.5. Next, the DCFH‐DA probe was added to the bacterial suspension and incubated at 37 °C for 0.5 h, with intermittent mixing every 5 min. The drug treatment was the same as above and incubated at 37 °C for 1 h. Finally, 200 µL of the bacterial suspension was added to the microplate and the fluorescence intensity was measured using a multifunctional microplate reader with an excitation wavelength of 488 nm and emission wavelength of 525 nm. All tests were performed in triplicate.

### ΔpH Measurement

TN‐P128 in the exponential growth phase were collected and washed three times with PBS buffer after centrifugation. The bacteria were then resuspended in buffer to form a bacterial suspension with OD_600_ = 0.5. The suspension was diluted ten‐fold. BCECF‐AM was added to the bacterial suspension at a final concentration of 10 µmol L^−1^ and incubated at 37 °C for 30 min. The drug treatment was the same as above and incubated at 37 °C for 1 h. Subsequently, the fluorescence intensity was measured using a multifunctional microplate reader with an excitation wavelength of 500 nm and an emission wavelength of 522 nm. All tests were performed in triplicate.

### Ethical Approval

This study was carried out in accordance with the recommendations of the ethical guidelines of Anhui Agricultural University. All animal experimental protocols were revised and approved by the Anhui Agricultural University Institutional Animal Ethics Committee (2022c057).

### Safety Evaluation

Briefly, 100 µL of PBS solution was added to the first nine columns of a 96‐well plate, and 100 µL of 0.2% Triton X‐100 as a positive control in the last column. 100 µL of PLU (256 µg mL^−1^) was added to column 6, 100 µL of MEV to column 7, and 100 µL of FMEV to column 8. Column 9 was kept as a negative control. Next, 100 µL of 8% sheep red blood cell suspension was added to each well of the 96‐well plate and incubated at 37 °C for 1 h. The red blood cells were removed and the absorptive value of the resulting supernatant was determined at 576 nm. The rate of hemolysis was calculated using the following formula:

(3) $$\begin{array}{cc} \mathrm{Hemolysis}\;\left(\%\right) & =\left(OD_{576}\mathrm{sample}-OD_{576}\mathrm{blank}\right)/\\ & \;\left(OD_{576}\mathrm{Triton}-OD_{576}\mathrm{blank}\right)\times 100\% \end{array}$$

### Mouse Model of Infection

The sample size was selected according to the initial infection test (mouse model *n* = 7). Female ICR mice were randomly housed in either treated or control cages. Mouse peritonitis–sepsis model: seven female mice in each group were injected intraperitoneally with 4.0 × 10^7^ CFU *E. coli* B2 suspension. At 1 h after infection, mice were infused with PBS, PB (8 mg kg^−1^), PLU (16 mg kg^−1^), PB + PLU (8 mg kg^−1^ + 16 mg kg^−1^), and FMEV (PB: 8 mg kg^−1^ + PLU: 16 mg kg^−1^). After the death of infected mice, different organs including the heart, liver, spleen, lung, and kidney were harvested and subjected to bacterial vehicle and histological analysis with sterile PBS homogenate within 48 h. The surviving mice were euthanized by cervical dislocation after 48 h infection. Each suspension was sequentially diluted for plated plate colony counting. The heart, liver, spleen, lung, and kidney samples were stained with hematoxylin and eosin for pathological examination.

### Statistical Analysis

Statistical analysis was performed using GraphPad Prism 8.0 (GraphPad Software, Inc.). Sample size (*n*) for each statistical analysis was indicated and experimental data were represented as mean ± standard deviation (s.d.). Tukey's multiple comparison test and the unpaired student's *t*‐test were used for all statistical analyses except specific experiments. Differences were considered significant at *p < 0.05, **p < 0.01, or ***p < 0.001. Not significant (*p* > 0.05) was indicated with ns.

## Conflict of Interest

The authors declare no conflict of interest.

## Supporting information



Supporting Information

## Data Availability

The data that support the findings of this study are available on request from the corresponding author. The data are not publicly available due to privacy or ethical restrictions.
