# Supplementary material for: A Milk Extracellular Vesicle‐Based Nanoplatform Enhances Combination Therapy Against Multidrug‐Resistant Bacterial Infections
Source: Adv Sci (Weinh). 2024 Dec 25;12(7):2406496. doi: 10.1002/advs.202406496 (PMC11831456; doi:10.1002/advs.202406496)
Supplement: Supplementary file 1 — Supporting Information [file ADVS-12-2406496-s001.docx]

Supporting Information

**A Milk Extracellular Vesicle-Based Nanoplatform Enhances Combination Therapy Against Multidrug-Resistant Bacterial Infections**

Shaoqi Qu^*^, Shuo Yang, Qingjun Xu, Mengying Zhang, Feng Gao, Yongning Wu^*^, Lin Li^*^

Prof. S. Qu, S. Yang, Q. Xu, M. Zhang, F Gao, Prof. Y. Wu, Prof. L. Li

Animal-Derived Food Safety Innovation Team

College of Veterinary Medicine

Anhui Agricultural University

Hefei 230036, China

Email: [sqq@ahau.edu.cn](mailto:sqq@ahau.edu.cn); wuyongning@cfsa.net.cn; [lilin@ahau.edu.cn](mailto:lilin@ahau.edu.cn).

Prof. Y. Wu,

Research Unit of Food Safety

Chinese Academy of Medical Sciences (No. 2019RU014)

NHC Key Laboratory of Food Safety Risk Assessment

China National Center for Food Safety Risk Assessment (CFSA)

Beijing 100022, China.

**Keywords**: multidrug resistance, bacteria, polymyxin, plumbagin, milk extracellular vesicles.

**Materials and methods**

**Time-kill curves**

According to the results of checkerboard assays, five TCMs that have the best-combined drug effect were selected. Time killing curve of PB, TCM and combination group were constructed. DMSO and ddH_2_O were used as a control group and solvent group, respectively. TN-P128 was cultured to the logarithmic phase and the bacterial content was adjusted to approximately 1.5×10^6^ CFU/mL. Bacterial suspensions were mixed with 1 MIC PB and corresponding TCM and then transferred into a 10 mL EP tube. After the liquid in the pipe had been adjusted to 5 mL by adding CAMHB broth, bacteria were cultured at 37 °C with shaking at 200 rpm for 24 h. 50 µL aliquots were removed at 0, 3, 6, 9, 12, and 24 h, and then mixed with 1000 µL CAMHB. Subsequently, bacterial suspensions were diluted in a tenfold series and inoculated on an LB solid medium. CFUs were calculated after incubation at 37 °C for 18 h. All experiments were replicated three times.

**Determination of the efflux pump**

TN-P128 bacteria in the exponential growth phase were collected by centrifugation at 4000 rpm for 10 min. Afterward, the bacteria were washed three times with PBS buffer, resuspended in buffer, and prepared to form a bacterial suspension with OD_600_=0.5. This suspension was then diluted 10-fold. To the bacterial suspension, EtBr was added to reach a final concentration of 5 µmol/L, and the mixture was thoroughly blended. We divided the bacterial suspension into four groups: control, PB (2 μg/mL), PLU (2 μg/mL), PB (2 μg/mL) + PLU (2 μg/mL), each with a total volume of 1 mL. Fluorescence intensity was measured using a multifunctional microplate reader with an excitation wavelength of 530 nm and an emission wavelength of 600 nm. All tests were performed in triplicate, and the raw data were analyzed using Prism software.

**Membrane fluidity assay**

TN-P128 bacteria in the exponential growth phase were collected by centrifugation at 4000 r/min for 10 min. Afterward, the bacteria were washed three times with PBS buffer, resuspended in buffer, and prepared to form a bacterial suspension with OD_600_=0.5. A membrane fluidity fluorescent probe (TMA-DPH) was added to the bacterial suspension to a final concentration of 10 µmol/L and fully mixed. The bacterial suspension was divided into four experimental groups: blank control, PB (2 μg/mL), PLU (2 μg/mL), PB (2 μg/mL) + PLU (2 μg/mL), total volume of 1 mL, incubation for 1 hour 37 ^o^C. Fluorescence intensity was measured using a multifunctional microplate meter excitation wavelength 360 nm and emission wavelength 440 nm.

**Protein and nucleic acid leakage assay**

TN-P128 bacteria in the exponential growth phase were collected and adjusted to a turbidity of 0.5 (1×10^8^ CFU/mL). The bacteria were divided into four experimental groups and incubated at 37°C for 2, 4, 6, 8, 10, 12 and 24 h. After each incubation period, the mixture was centrifuged at 5000×g for 4 min, and 100 μL of the supernatant was absorbed into a 96-well plate. The protein concentration was determined using the Bradford assay, and the absorption values at 260 nm were measured using a microplate reader.

**Intracellular accumulation assay of PLU**

TN-P128 bacteria, in their exponential growth phase, were collected and evenly divided into two experimental groups. One group was treated with PB (8 μg/mL), while the other group received both PB (8 μg/mL) and PLU (16 μg/mL). After the incubation at 37°C for 4 h, a 20 mL sample of the bacterial culture was taken and centrifuged at 3000 rpm for 10 minutes. The supernatant was removed, and the pellet was washed three times with PBS. The remaining bacterial pellet was resuspended in 1 mL of acetonitrile and sonicated to break open the cells. The absorbance was measured at 410 nm using a spectrophotometer to determine the PLU concentration, according to a previously established standard curve.

**Determination of PB in FMEV**

The analysis was conducted using an Agilent Infinite Laboratory Hole Shell 120 Aq-C18 column (250 x 4.6 mm; 5 μm, Agilent Technologies, Santa Clara, CA, USA). For the buffer preparation, 4.46 g of anhydrous sodium sulfate was dissolved in 900 mL of ultrapure water, the pH was adjusted to 2.3, and the volume was brought up to 1000 mL with ultrapure water. This was followed by filtration of the water phase. The mobile phase consisted of acetonitrile, which was filtered through an organic phase filter membrane. The acetonitrile to buffer ratio was set at 20:80. The column was operated at a flow rate of 1.0 mL/min and a temperature of 35°C. UV absorbance for both fingerprinting and quantitative analysis was monitored at 215 nm.

**Determination of intestinal tight junctions after citric acid feeding**

In vivo experiment: The sample size was selected based on the initial feeding test (n = 3 for the mouse model). Mice were randomly housed in the treated cages. Mouse citric acid feeding model: three female mice in each group were fed with PBS, 200 μL (4, 8, 16 g/L) citric acid and euthanized after 4 h of cervical dislocation. Remove fresh or -80 ^o^C frozen tissue, add 1 mL Total RNA Extractor per 30-50 mg of tissue and homogenate with a homogenizer. After lysis, the samples or homogenates were left at room temperature for 5-10 min to allow the complete separation of the nuclear protein from the nucleic acid. 0.2 mL of chloroform was added to vigorously shake for 15 s at room temperature. The samples were centrifuged at 12,000 rpm for 10 min at 4 ^o^C. The upper aqueous phase was transferred to a clean centrifuge tube, adding an equal volume of isopropanol, mixed well, and placed at room temperature for 20 min. 4 ^o^C was centrifuged at 12,000 rpm for 10 min and the supernatant was discarded. The precipitate was washed by adding 1 mL of 75% ethanol. The sample was centrifuged at 12,000 rpm for 3 min at 4 ^o^C and the supernatant was discarded. And they were dried at room temperature for 5-10 min. Between 30-50 μL RNase-free ddH_2_O was added to fully dissolve the RNA. The resulting RNA and protein solutions were stored at -80 ^o^C for subsequent testing.

The protein extract stored by -80 ^o^C was verified for WB of related tight junction protein ZO-1 and Occludin; the RNA extract stored by -80 ^o^C was sent to Shanghai Biotech for qPCR to determine the expression of related tight junction protein gene Claudin, ZO-1, ZO-2 and Occludin.

In vitro experiments: IEC-6 cells were harvested via centrifugation at 2000 rpm for 5 minutes. Citric acid at concentrations of 1 and 2 g/L was then added, and the samples were incubated at 37°C for 2 hours. Following the incubation period, the cells were collected through centrifugation at 2000 rpm for 5 minutes, and RIPA buffer was added to lyse the cells. The lysate was mixed thoroughly and chilled on ice for 30 minutes. Post-lysis, the mixture was subjected to centrifugation at 4°C (10,000 rpm, 10 minutes) to separate the supernatant. The protein solutions extracted were subsequently stored at -80°C for further analysis.

**Safety evaluation**

To assess the systemic toxicity of MEV and FMEV, female ICR mice aged 6-8 weeks and weighing 20.0 ± 0.5 g were utilized (obtained from Qinglong Mountain Animal Farm, Nanjing, China; Animal License No. SYXK2018-0049). The mice were housed under controlled conditions of 23 ± 2 °C, a 12-hour light/dark cycle, and 50 ± 5% relative humidity, with free access to distilled water and standard rodent chow. Following adaptive feeding, the mice were randomly assigned to one of three groups: PBS, MEV (6mg/kg) and FMEV (6 mg/kg) groups. After 24 h, blood was collected via enucleation, and the serum was analyzed using a biochemical analyzer (Mindray BC-5000VET).

For in vitro safety assessments, we utilized passaged RAW 264.7 and HepG-2 cells. We transferred 100 μL of a homogeneous cell suspension into each well of a 96-well plate, aiming for a density of roughly 1×10^5^ cells/well. After a 12-hour incubation period to allow cells to fully adhere, FMEV of mixed PB (8 µg/mL) and PLU (16 µg/mL) was introduced into the cells at different final concentrations (1, 5, 10, 25, 50, and 100 μg/mL) and co-cultured for 24 hours. Subsequently, we added 10 μL of CCK-8 solution to each well and incubated for an additional 30 minutes. Finally, we measured the absorbance of the mixture at 450 nm using a microplate reader.

Figures


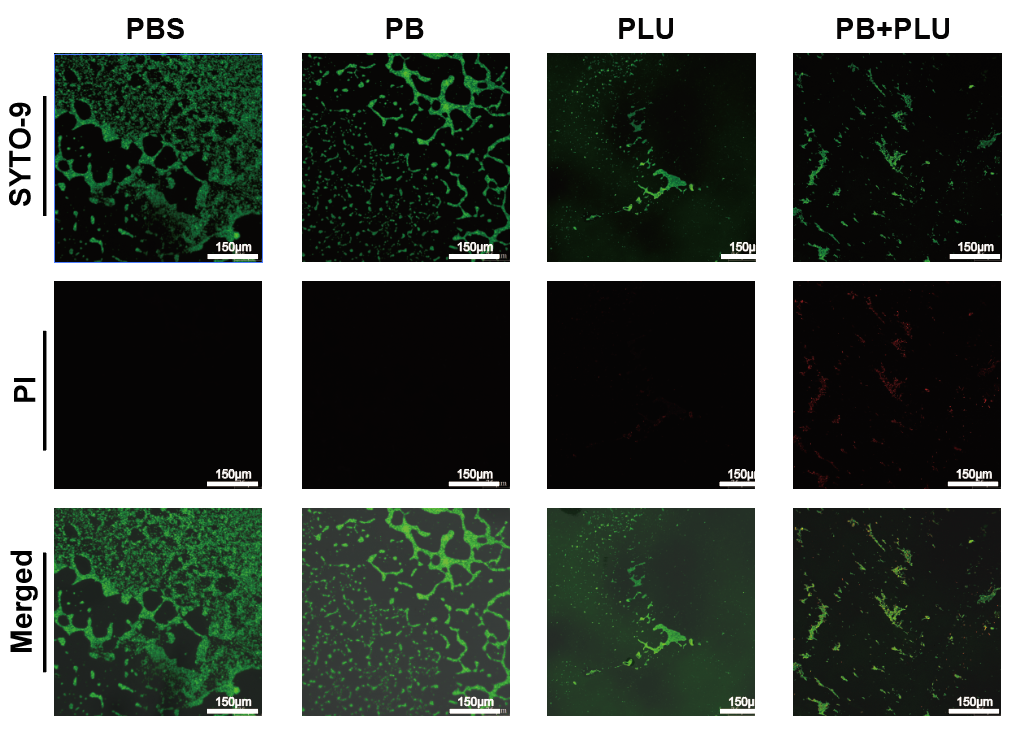


**Fig S1. The combination of PLU and PB is used to effectively eliminate the biofilm of TN-P128.** Representative confocal images of TN-P128 under the different treatments. Viable bacterial cells were stained green by SYTO9, whereas dead cells were stained red by PI.


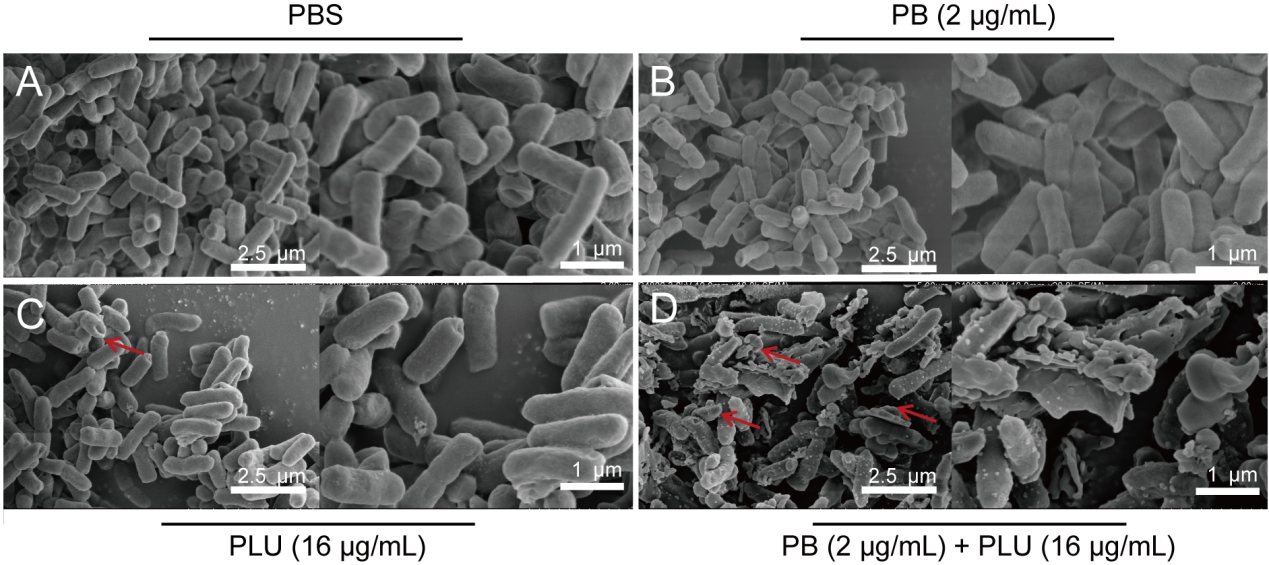


**Fig S2. Representative SEM images of *E. coli* B2 treated with PB and PLU.** Bacteria were treated with PBS, PB (2 µg/mL), PLU (16 µg/mL), and PB + PLU (2 µg/mL + 16 µg/mL), respectively.


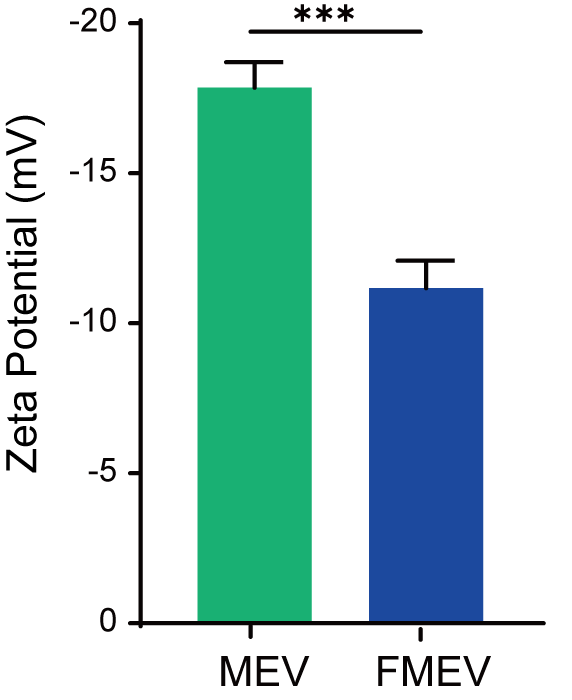


**Fig S3.** **Determination of the zeta potential of MEV and FMEV.** n = 3 independently and identically performed experiments (mean ± s.d.). ****p* < 0.001 by *t*-test.

**
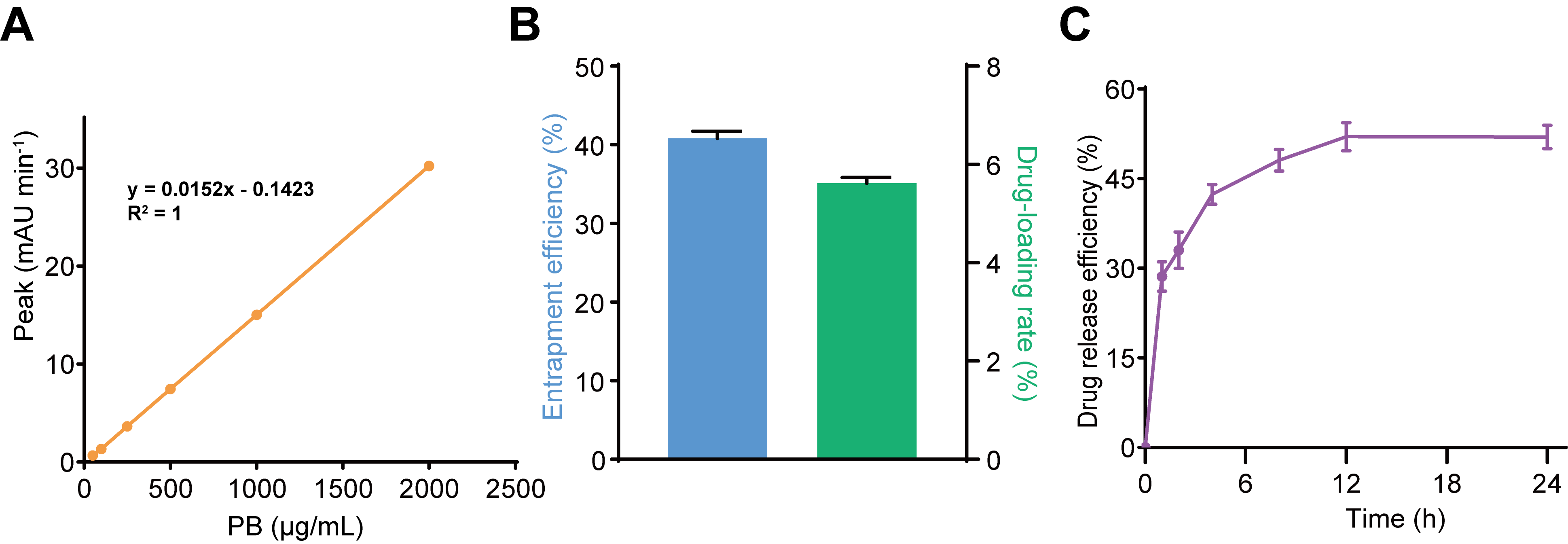
Fig S4. Characterization of drug loading.**

(A) A linear regression equation was obtained using high-performance liquid chromatography for PB concentrations ranging from 50-2000 µg/mL.

(B) Determination of the drug loading and encapsulation efficiency of PB in FMEV.

(C) The release profile of PB from FMEV after 24 hours at 37℃, under neutral conditions, showed a rapid drug release within the first 1-4 hours.


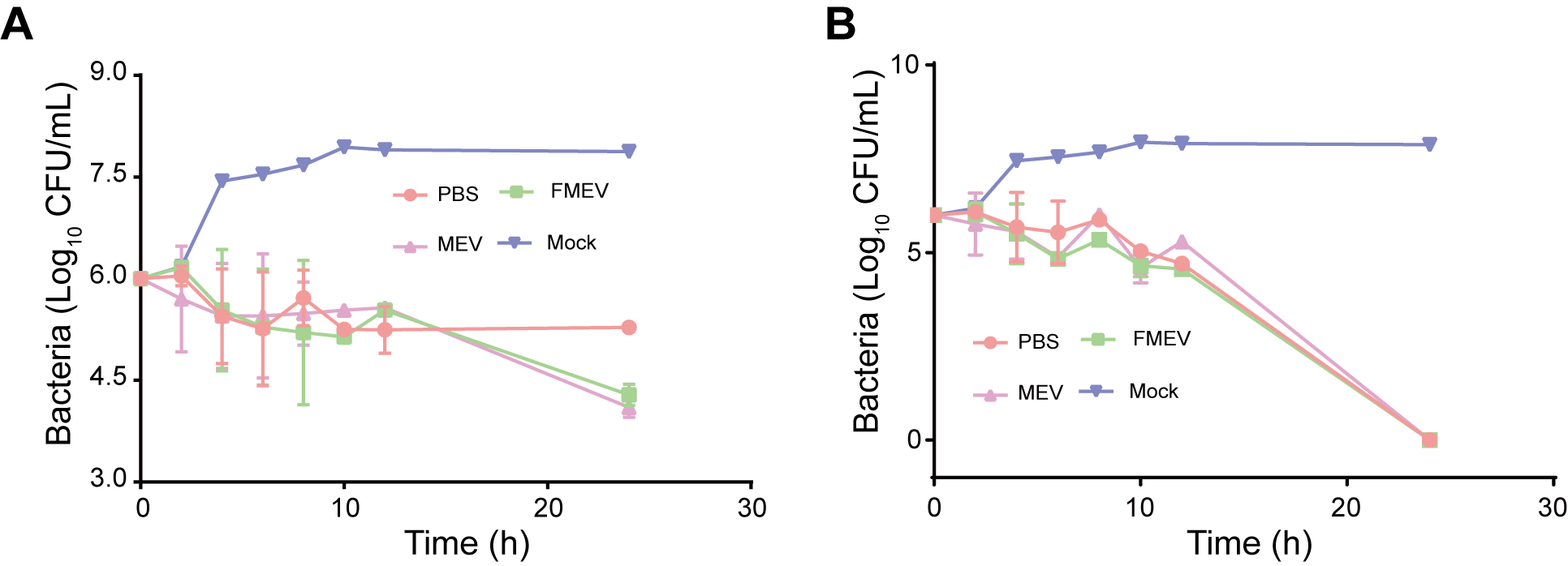


**Fig S5.** Time-kill curves of TN-P128 at an exponential phase in the presence of FMEV and the combination of PB with PLU at levels of (A) 8 µg/mL + 16 µg/mL and (B) 8 µg/mL + 32 µg/mL.


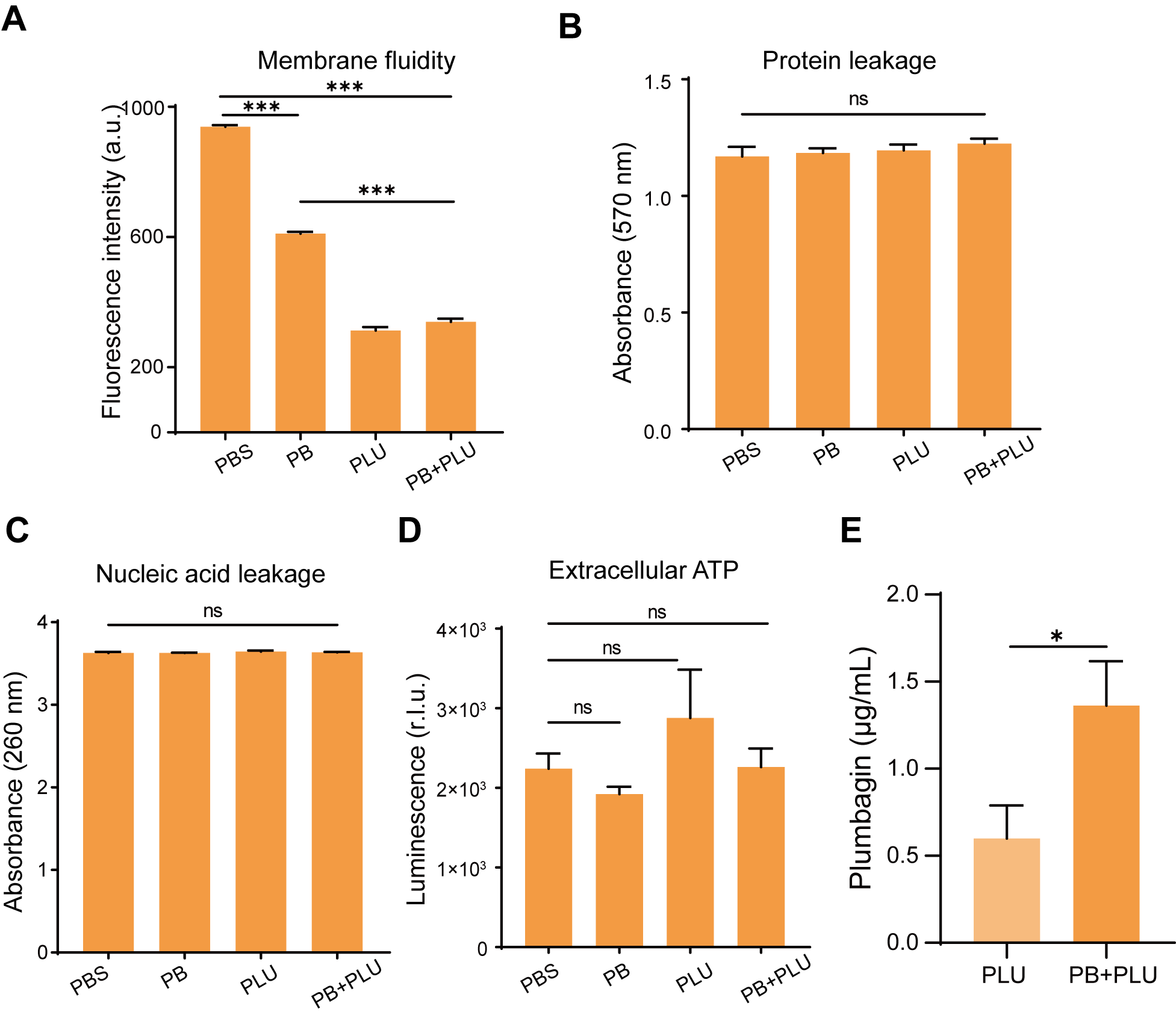


**Fig S6. Mechanism of PLU in combination with PB against TN-P128.**

**(A)** PLU in combination with PB significantly reduced the cell membrane fluidity of TN-P128.

**(B)** When PLU was used in combination with PB, no protein leakage occurred in TN-P128.

**(C)** When PLU was used in combination with PB, there was no nucleic acid leakage in TN-P128.

**(D)** PLU combined with PB had no significant effect on the extracellular ATP levels of TN-P128.

**(E)** Combined use of PB and PLU accelerates PLU accumulation in bacteria. n = 3 independently and identically performed experiments (mean ± s.d.). Significance was determined by Tukey's multiple comparisons test following one-way ANOVA. ns, not significant. ****p* < 0.001.


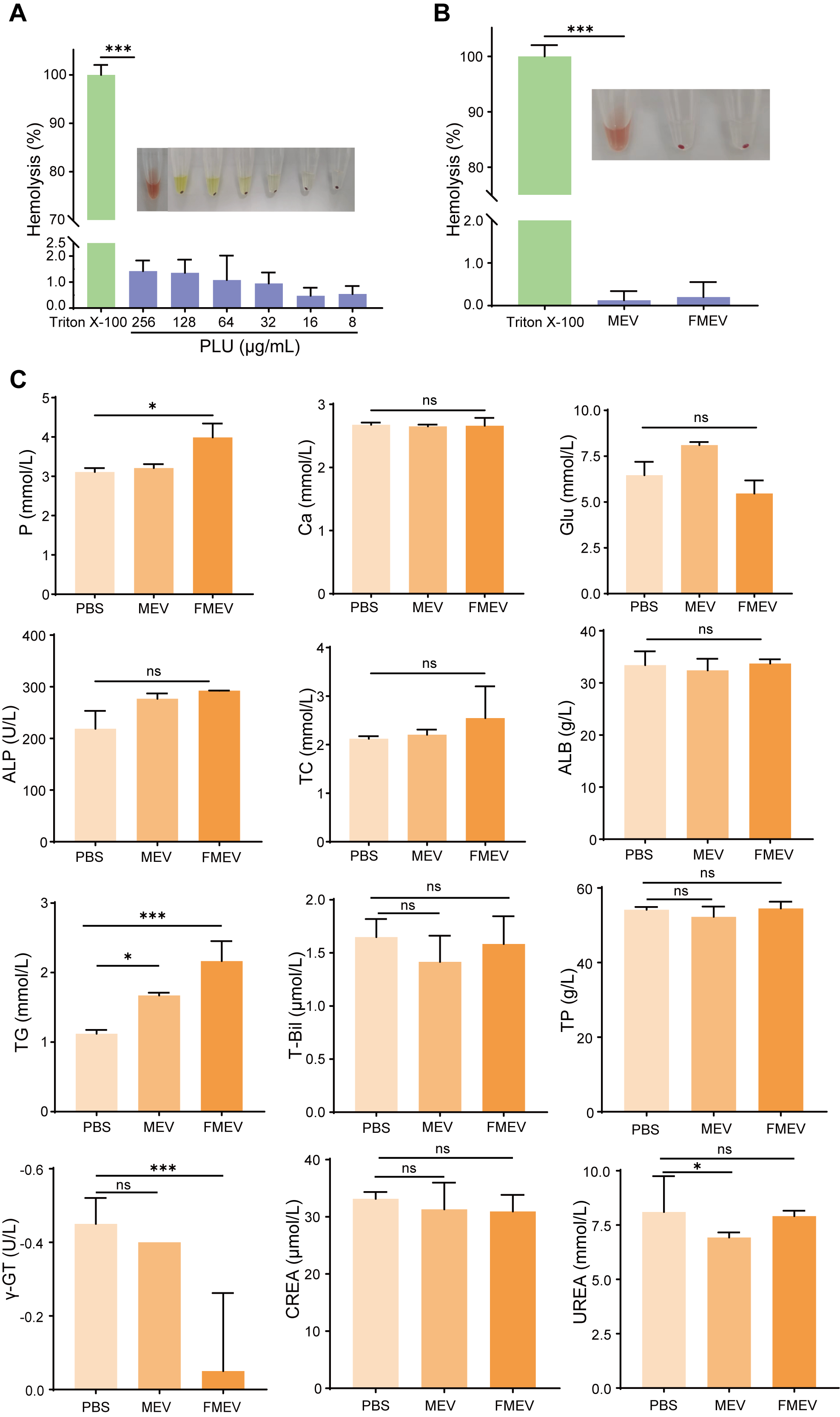


**Fig S7. Safety evaluation of MEV and FMEV.**

**(A)** Hemolytic activity of PLU to the red blood cells of sheep. Different concentrations of PLU (2-256 µg/mL) had no hemolytic toxicity.

**(B)** Hemolytic activity of MEV nor FMEV to the red blood cells of sheep.

**(C)** Evaluation of biochemical parameters in mice treated with MEV and FMEV. P: Serum phosphorus; Ca: Calcium serum; Glu: Serum glucose; ALP: Serum alkaline phosphatase; TC: Total cholesterol; ALB: Albumin; TG: Triglyceride; T-Bil: Total bilirubin; TP: Total protein; γ-GT: Serum γ-glutamy ltransferase; CREA: Serum creatinine; UREA: Serum urea. n = 3 independently and identically performed experiments (mean ± s.d.). Significance was determined by Tukey's multiple comparisons test following one-way ANOVA. ns, not significant. **p* < 0.05 and ****p* < 0.001.


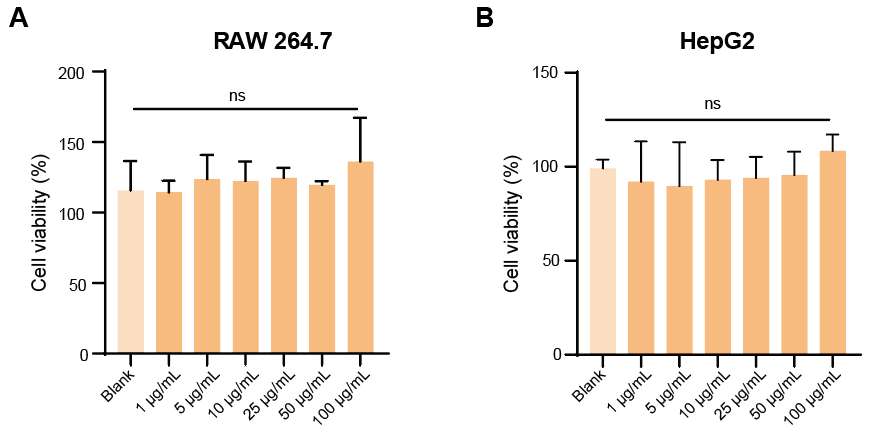


**Fig S8.** **FMEV was co-incubated with the cells without cytotoxicity.**

Cell viability was evaluated using FMEV concentrations from 1 to 100 µg/mL (at 1, 5, 10, 25, 50, and 100 μg/mL intervals) after 24 hours of co-culture with RAW 264.7 (A) and HepG2 (B) cells. The experiments were independently and identically performed three times, and the results are presented as mean ± s.d. Significance was determined by Tukey's multiple comparisons test following one-way ANOVA. ns, not significant.


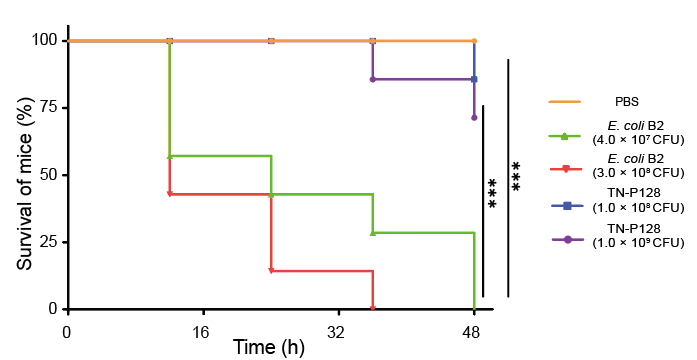
**Fig S9. Survival curves of mice with different administration doses of *E. coli* B2 and** ***S.* Typhimurium TN-P128.**

*E. coli* B2 (4.0 × 10^7^ CFU) killed all mice within 48 h, and *S.* Typhimurium TN-P128 (1.0 × 10^9^ CFU) failed to kill half of them within 48 h. n=7，*P* values were determined using the two-sided, log[rank] (Mantel–Cox) test. ****p* < 0.001.


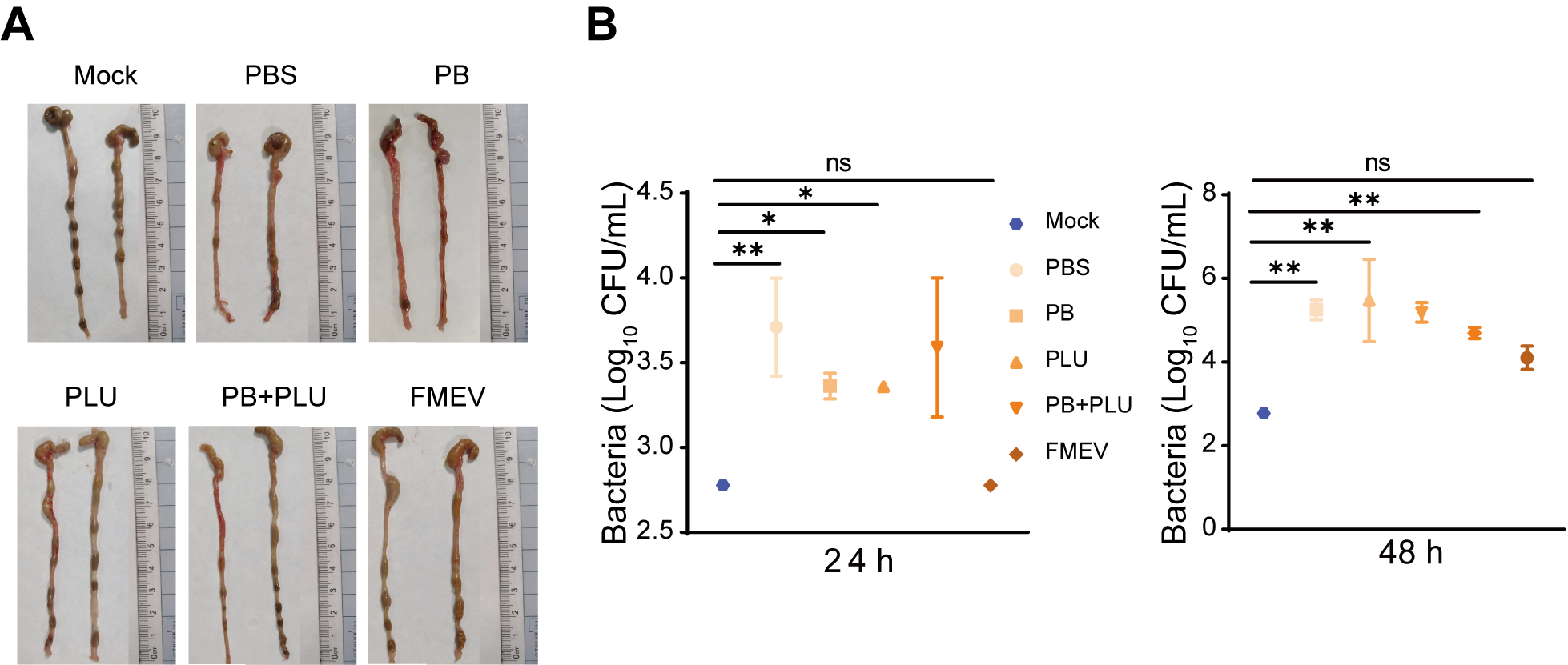


**Fig S10. FMEV improved the therapeutic effect of peritonitis infection model in mice.**

**(A)** The length of colon of mice in different groups (n=7).

**(B)** The bacterial load of *E. coli* B2 in feces collected from mice after the treatment for 24 h and 48 h were determined. Significance was determined by Tukey's multiple comparisons test following one-way ANOVA. ns, not significant. **p* < 0.05 and ***p* < 0.01.


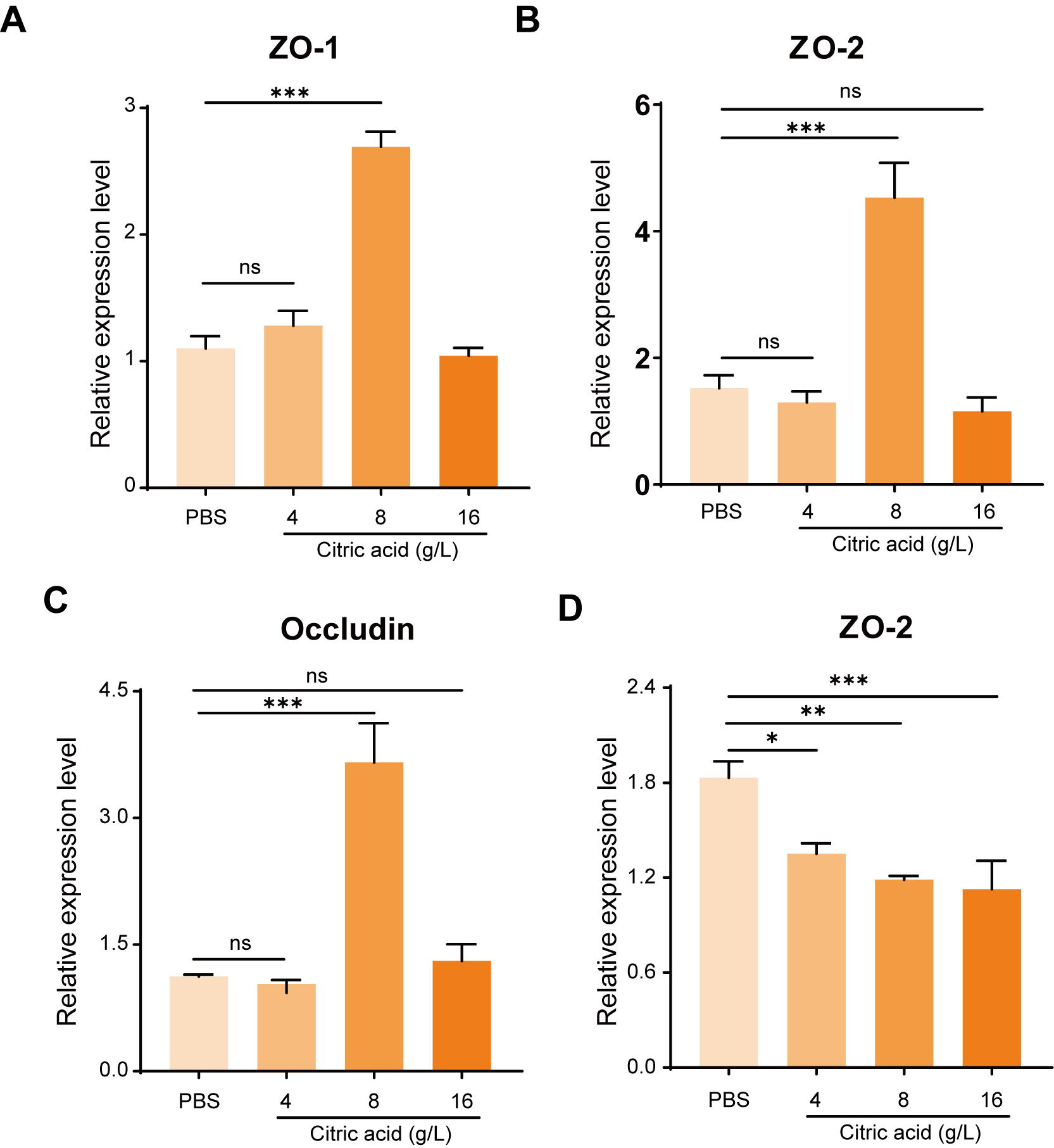


**Fig S11. Citric acid destroyed the tight junction of mouse intestinal epithelium.**

**(A-C)** Relative expression and quantification of tight junction related proteins Claudin-10 (A), Claudin-7 (B), Claudin-2 (C), ZO-1 (D), and Occludin (E) genes in duodenum of mice treated with FMEV. The expression of genes related to tight junction was decreased.

**(D)** Relative expression and quantification of tight junction related protein ZO-2 genes in jejunum of mice treated with FMEV. The expression of genes related to tight junction was decreased. n = 3 independently and identically performed experiments (mean ± s.d.). Significance was determined by Tukey's multiple comparisons test following one-way ANOVA. ns, not significant. **p* < 0.05, ***p* < 0.01 and ****p* < 0.001.


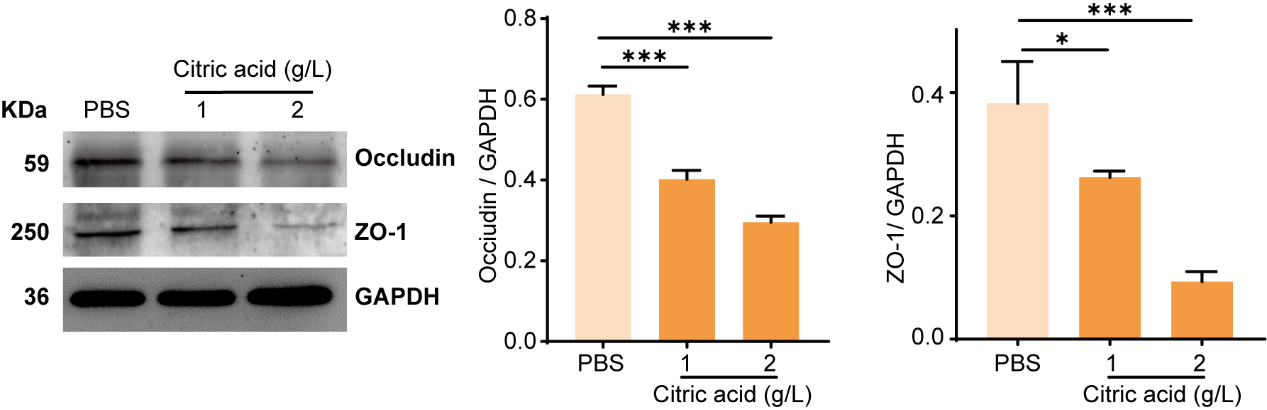


**Fig S12. The mechanism of citric acid-mediated reversible opening of tight junctions to enhance transcellular transport.**

The relative expression of tight junction relative proteins ZO-1 and Occludin in citric acid-treated IEC-6 cells was detected and quantitatively analyzed by immunoblotting. The secretion of ZO-1 and Occludin proteins from IEC-6 cells was significantly decreased by citric acid treatment, which indicated that citric acid promoted the opening of TJs. n=3 independently and identically performed experiments (mean ± s.d.). Significance was determined by Tukey's multiple comparisons test following one-way ANOVA. **p* < 0.05 and ****p* < 0.001.

**Tables**

**Table S1.** Antibiotic susceptibility results for TN-P128

|  | MIC (µg/mL) | | | |
| --- | --- | --- | --- | --- |
| Antibiotics | *E. coli* ATCC25922 | *S.* Typhimurium ATCC13311 | TN-P128 | *E. coli* B2 |
| Ampicillin (AMP) | 2 | 0.5 | 0.5 | - |
| Ceftazidime (CAZ) | 0.12 | 0.25 | 0.25 | - |
| Meropenem (MEM) | 0.25 | 0.01 | 0.02 | - |
| Kanamycin (KAN) | 2 | 2 | 4 | - |
| Spectinomycin (SPE) | 16 | 32 | 64 | - |
| Tetracycline (TET) | 0.5 | 2 | 8 | - |
| Doxycycline (DOX) | 0.5 | 1 | 4 | - |
| Tegacyclin (TGC) | 0.25 | 0.25 | 0.25 | - |
| Polymyxin B (PB) | 1 | 0.5 | 128 | 8 |
| Tulathromycin (TUL) | - | 4 | 32 | - |
| Gamithromycin (GAM) | - | 8 | 64 | - |
| Ciprofloxacin (CIP) | 0.08 | 0.01 | 0.02 | - |
| Enrofloxacin (ENR) | - | 0.01 | 0.02 | - |
| Sulfafurazole (SF) | 16 | 64 | 64 | - |
| Florfenicol (FFC) | - | 0.25 | 32 | - |
| Mequindox (MEQ) | - | 0.25 | 1 | - |
| Apramycin (APR) | - | 4 | 8 | - |

**Table S2.** Classification results of traditional Chinese medicine.

| Compound | Compound type |
| --- | --- |
| Chalcone | Flavonoids |
| Baicalein |  |
| Puerarin |  |
| Berberine | Alkaloid |
| Fibraurea |  |
| Matrine |  |
| Resveratrol | Polyphenols |
| Gallic acid |  |
| Plumbapin | Naphthoquinone |
| Malol | Pentacyclic triterpene compound |
| Artemisinin | Sesquiterpene lactone |
| Capsaicin | Capsaicinoids |
| Magnolol | Lignans |

**Table S3.** Individual TCM drug susceptibility results of TN-P128

| **Compound** | **MIC (µg/mL)** | **Compound** | **MIC (µg/mL)** |
| --- | --- | --- | --- |
| Plumbapin (PLU)  Capsaicin (CAP)  Baicalein (BAI)  Malol (MAL)  Matrine (MAT)  Artemisinin (ART)  Chalcone (CHA) | 16  512  4096  8192  8192  16384  16384 | Forsythoside B (FIB)  Magnolol (MAG)  Berberine (BER)  Gallic acid(GAL)  Resveratrol (RES)  Puerarin (PUE) | >5120  80  >2560  640  2560  65536 |

**Table S4.** The FIC of TCM monomer under the treatment of PB for TN-P128

| Compounds | FIC (µg/mL) | FICI |
| --- | --- | --- |
| Polymyxin B (PB) | 4 | 0.281 |
| Plumbapin (PLU) | 4 |  |
| Polymyxin B (PB) | 2 | 0.141 |
| Capsaicin (CAP) | 64 |  |
| Polymyxin B (PB) | 8 | 0.125 |
| Matrine (MAT) | 512 |  |
| Polymyxin B (PB) | 0.25 | 0.064 |
| Chalcone (CHA) | 1024 |  |
| Polymyxin B (PB) | 2 | 0.031 |
| Resveratrol (RES) | 40 |  |
| Polymyxin B (PB) | 8 | 0.125 |
| Magnolol (MAG) | 5 |  |

**Table S5.** Results of the combination of PLU and PB against E. coli B2

| Compound | MIC (µg/mL) | FIC (µg/mL) | FICI |
| --- | --- | --- | --- |
| Polymyxin B (PB) | 8 | 2 | 0.375 |
| Plumbapin (PLU) | 128 | 16 |  |

## Table S6. Drug concentration before and after FMEV loading

| **Compound** | **Initial concentration** | **Loaded concentration** |
| --- | --- | --- |
| PB | 70.0 μg/mL | 28.6 μg/mL |
| PLU | 90.0 μg/mL | 77.4 μg/mL |

**Table S7.** Primer sequence for identification of *Salmonella* Typhimurium

| **Primer name** | **Sequence direction（5'→3'）** |
| --- | --- |
| *invA*-F  *invA*-R  *fljB*-F  *fljB*-R  *fliC*-F  *fliC*-R | GTGAAATTATCGCCACGTTCGGGCAA  TCATCGCACCGTCAAAGGAACC  CAACGGCGTGAAAGTCCTGG  GCACCAGTAAAGCCACCAAT  GTCTGGATACGCTGAATGTG  ACCTCACCGTTCGTCTTATC |
